# Reduced reliance on the trace element selenium during evolution of mammals

**DOI:** 10.1186/gb-2008-9-3-r62

**Published:** 2008-03-31

**Authors:** Alexey V Lobanov, Dolph L Hatfield, Vadim N Gladyshev

**Affiliations:** 1 Department of Biochemistry, University of Nebraska, Lincoln, NE 68588, USA; 2 Section on the Molecular Biology of Selenium, Laboratory of Cancer Prevention, National Cancer Institute, National Institutes of Health, Bethesda, MD 20892, USA

## Abstract

**Background:**

Selenium (Se) is an essential trace element that occurs in proteins in the form of selenocysteine (Sec). It is transported throughout the body in the form of Sec residues in Selenoprotein P (SelP), a plasma protein of unclear origin recently proposed as an experimental marker of dietary Se status.

**Results:**

Here, we report that the amino-terminal domain of SelP is distantly related to ancestral bacterial thiol oxidoreductases of the thioredoxin superfamily, and that its carboxy-terminal Se transport domain may have originated in early metazoan evolution by *de novo *accumulation of Sec residues. Reconstruction of evolutionary changes in the Se transport domain indicates a decrease in Sec content of SelP specifically in the mammalian lineage via replacement of Sec with cysteine (Cys). Sec content of mammalian SelPs varies more than two-fold and is lowest in rodents and primates. Compared to mammals, fish show higher Sec content of SelP, larger selenoproteomes, elevated SelP gene expression, and higher levels of tissue Se. In addition, mammals replaced Sec with Cys in several proteins and lost several selenoproteins altogether, whereas such events are not found in fish.

**Conclusion:**

These data suggest that evolution from fish to mammals was accompanied by decreased use of Sec and that analyses of SelP, selenoproteomes and Sec/Cys transitions provide a genetic marker of utilization of this trace element in vertebrates. The evolved reduced reliance on Se raises questions regarding the need to maximize selenoprotein expression by Se dietary supplements in situations when pathology is not imminent, a currently accepted practice.

## Abstract 2

Evolution from fish to mammals was accompanied by decreased use of selenocysteine, raising questions about the need for selenium dietary supplements when pathology is not imminent.

## Background

Several trace elements are essential micronutrients in humans and animals, but why some organisms utilize certain trace elements to a greater extent than others is not understood. It is also unknown how trace elements were utilized by extinct organisms, how the utilization changed during evolution, and how this affected their current use. These questions are not only important in addressing the roles trace elements played and continue to play in biology, but also have important implications with regard to human health, animal husbandry and veterinary practice. Dietary supplementation involving several trace elements, vitamins and other biofactors are an accepted practice in human and animal health care [1,2]. The Food and Nutrition Board of the National Research Council and the National Academy of Sciences, USA, set recommended dietary allowances (RDA), the estimated daily amount of a substance thought to be necessary for maintenance of good health. Trace elements are prominently featured in these reports as well as in labels on common foods.

One of the trace elements, selenium (Se), represents a particularly interesting case. It is used in proteins in the form of selenocysteine (Sec), the 21st naturally occurring amino acid in the genetic code [3-5]. Sec differs from cysteine (Cys) by a single atom (Se versus S). Sec is encoded by the UGA codon and its co-translational insertion into protein requires an RNA structure known as the SECIS (for SEC Insertion Sequence) element. Selenoproteins are important antioxidant enzymes and also have other redox functions [6]. Several human disorders have been associated with Se deficiency, such as Keshan disease, Kashin-Beck disease and myxedematous endemic cretinism (OMIM identifiers 606210 and 601484) [7,8]. The RDA for Se is based on the amount required to maximize the synthesis of glutathione peroxidase (GPx)3 [9]. Current US dietary recommendations for Se for both men and women are 55 μg/day [10]. Although the normal intake of Se by eating food is sufficient to meet the RDA for this essential nutrient everywhere in the US, approximately 20-30% of Americans consume multivitamin/mineral supplements daily [11], and a significant part of them contain Se.

We have previously analyzed the occurrence of selenoproteins and Se utilization traits in prokaryotes and found that only 20% of these organisms utilize Sec [12]. Sec utilization in eukaryotes is also sporadic, and certain eukaryotes, such as fungi, vascular plants and some insects, do not utilize it [13]. However, in mammals, Se is an essential trace element. In mice, embryonic lethality is caused by disruption in several selenoprotein genes, such as those encoding thioredoxin reductase (TR)1 and TR3, and GPx4 [14-16], and several additional selenoproteins were implicated in protection against disease [17,18]. Previously analyzed mammalian selenoproteomes consist of 24-25 selenoproteins, whereas lower eukaryotes and prokaryotes mostly have very few of these proteins (for example, only 4 selenoproteins have been found in *Plasmodium *and 3 in *Escherichia coli*) [19-22]. These observations established Se genetics and genomics as a useful evolutionary model system to address the issues of evolutionary changes in utilization of this trace element as well as the use of Se by living and extinct organisms.

In the current study, we report on the use of Selenoprotein P (SelP), selenoproteomes and Sec/Cys transitions as a genetic marker to assess the status and evolutionary trends in Sec and Se utilization. SelP accounts for the major pool of plasma Se [23,24]. Human, mouse and rat SelPs have 10 Sec residues [19]. The high content of Se in these proteins has led to the hypothesis that SelP acts as a transport protein and is responsible for Se delivery to various organs and tissues [25]. Recent studies support this idea [26-29]. In mammals, SelP is primarily synthesized in the liver and delivers Se to kidney, brain, testes, and other organs. Isolated hepatic SelP deficiency does not alter brain Se levels [30], yet brain and in particular hippocampal Se levels were lowered by disruption of the gene encoding SelP, but not by Se deficiency [31]. SelP has two functional SECIS elements in the 3' untranslated region (UTR) [32], whereas a single SECIS element was reported in all other known selenoprotein genes. The first UGA codon in SelP is served primarily by a relatively inefficient distal SECIS element, whereas the other SECIS element is responsible for insertion of all other Sec residues [33]. The high Sec content of the carboxy-terminal Sec-rich domain of SelP was shown to be required for the role of this protein in Se transport [34].

SelP was recently proposed as an experimental marker of Se utilization in humans that could be more accurate than the currently used GPx3 marker [9]. It was found that while GPx3 expression is saturated by the current RDA for Se, the specific amount of Se needed to achieve maximal expression of SelP is approximately 100 μg/day. Interestingly, both SelP and GPx3 studies were based on the premise that saturated expression of these proteins is required for optimal health and that even partial deficiency in any selenoprotein may be detrimental. However, in the current study, genomics analyses suggested a trend toward reduced utilization of Se in mammals, which could be seen at the level of both Sec content of SelP and unidirectional Sec/Cys transitions in vertebrate selenoproteins. These data are discussed with regard to the currently accepted practice of maximizing selenoprotein expression by dietary supplements.

## Results and discussion

### Occurrence of SelP homologs in organisms from nematodes to mammals

SelP was previously identified in fish, birds, and mammals. We carried out PSI-BLAST analyses with known SelP sequences as queries to search protein databases for distant SelP homologs. The sequences identified served as new queries in searches for SelP homologs in nucleotide sequence databases, including non-redundant, expressed sequence tag (EST), completed genome, whole genome shotgun (WGS), high throughput genome sequence and nucleotide trace databases. These searches identified SelP homologs in organisms from nematodes and primitive aquatic animals to mammals, suggesting that SelP evolved in an early metazoan lineage rather than in vertebrates as previously thought. However, several invertebrate animals characterized by completely sequenced genomes (for example, *Drosophila*) lacked SelP, suggesting that these organisms lost these proteins during evolution. One of the earliest metazoans, *Trichoplax adhaerens*, also lacked SelP, yet we detected at least 21 selenoproteins in this organism (data not shown).

We developed an additional approach to identify SelP sequences, wherein we searched genome and EST databases for occurrence of two proximal SECIS elements (Figure S1 in Additional data file 1). We screened all ESTs available in GenBank (March 2007), and the sequences upstream of two candidate SECIS elements were analyzed in three open reading frames for similarity to known proteins. This procedure yielded 32 full or partial non-redundant SelP sequences, most of which were of fish and mammalian origin. Only two additional sequences were detected, one of which, from the plant *Populus tremuloides*, could not be functional because higher plants lack selenoprotein genes, and the other sequence corresponded to *Carcinoscorpius rotundicauda *SelW containing one predicted SECIS in the open reading frame and the second in the 3'-UTR. We recently reported that coding region SECIS elements are functional in higher eukaryotes [35], but 3'-UTR structures are more efficient. Thus, two-SECIS mRNAs are a unique feature of SelP sequences, and the search for proximal SECIS elements can specifically recognize SelP in sequence databases. These data suggest that additional widely distributed selenoproteins containing many (for example, more than two) Sec are either extremely rare or do not exist.

### SelP has a thioredoxin-fold domain

**Figure 1:**
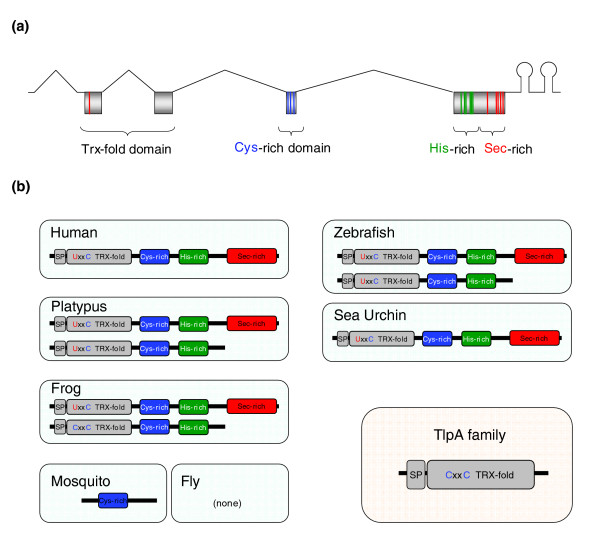
Domain organization of SelP and structures of SelP genes. **(a) **Domain organization of SelP sequences. Intron-exon structure of SelP is shown, and four domains are indicated. **(b) **Domain organization and homologous proteins. Fish (zebrafish is used as an example) and early mammals (for example, platypus) have two SelPs, a full-length protein SelPa and a shorter SelPb. Other mammals have only SelPa. Insects have either a short homolog that corresponds to exon 4 in mammalian SelP genes (for example, mosquito), or no homologs at all (fly). SP, signal peptide.

**Figure 2:**
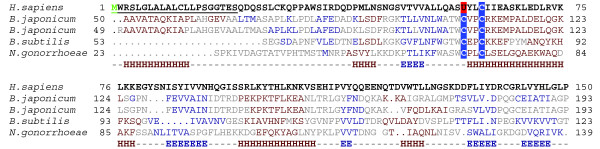
SelP is a thioredoxin fold-like protein. A multiple alignment of SelP and TlpA proteins is shown. Consensus secondary structure is shown below the sequences (helices are shown as H and strands as E). Helices are shown in brown and strands in blue. Sec and Cys in the UxxC motif are highlighted in red and blue, respectively. Starting Methionine is shown in green. Signal peptides are underlined.

Genomic analyses revealed that the human and mouse SelP genes consisted of five exons (Figure 1a), with the first exon corresponding to the 5' end of the 5'-UTR, exons 2-4 to the coding region, and exon 5 to the carboxy-terminal part of the protein and the 3'-UTR. Multiple alignment of SelPs (Figure S2 in Additional data file 1) revealed highly conserved sequences within the amino-terminal region (coded by exons 2-4), which had a single Sec. Conservation of carboxy-terminal sequences was low, and their Sec content varied significantly. Structural analyses of SelP sequences using 3D-Jury [36] revealed similarity of amino-terminal sequences (coded by exons 2 and 3) to thioredoxin fold proteins (Figure 2), and showed that the location of the UxxC motif in SelP corresponded to the CxxC motif in thioredoxins. This observation suggests a redox function of the amino-terminal domain. Further analysis showed that five proteins with J-scores of 50.20-71.60 (threshold value is 50) are structurally related to SelP, including thiol-disulfide interchange proteins TlpA and thiol-disulfide oxidoreductase ResA sequences. TlpA and ResA are bacterial protein disulfide reductases that play important roles in cytochrome c maturation and represent membrane anchored proteins with a thioredoxin domain containing a CxxC motif [37,38]. These observations further suggest a redox function of the amino-terminal domain of SelP.

Immediately downstream of the Trx-fold domain was a conserved region (coded by exon 4), which we designate as the Cys-rich domain (Figure 1b). This sequence, but not upstream or downstream SelP sequences, was observed in several insect genomes. Exon 5 was the largest exon in SelP genes and coded for the remainder of the SelP sequence, including His-rich and Sec-rich regions, and also included the 3'-UTR. SelP is known to occur in two forms, SelPa and SelPb, that differ by the presence of the Sec-rich region. Both forms have the His-rich region that mediates heparin binding and could account for the membrane binding properties of SelP [39].

In addition to the selenium transport function [26,28], SelP was shown to reduce phospholipid hydroperoxides in a cell-free *in-vitro *system [40]. This function may be mediated by the amino-terminal domain. An attractive possibility is that the amino-terminal domain serves as a redox partner for the carboxy-terminal Sec-rich region of SelP. For example, the amino-terminal domain could be responsible for keeping Sec residues in the oxidized state while the protein is in transit in the circulatory system, or for the reduction of Sec in SelP upon import of this protein into cells. Controlled oxidation of Sec residues to selenenylsulfides and diselenides may protect them against oxidation of Se to selenenic and further oxidized forms, which may lead to the loss of Se from SelP.

### Sec content of SelP sequences

In contrast to a single human SelP containing 10 Sec residues [41], zebrafish has 2 SelP isozymes [42], with SelPa and SelPb containing 17 and 1 Sec residue, respectively [19,43]. We examined the collection of SelP sequences derived from genomic, non-redundant and EST databases and found that the Sec content of SelP varied from zero to 28. Moreover, the Sec content of mammalian SelP varied more than two-fold; for example, dog SelP had 15 Sec residues, whereas guinea pig SelP had 7. Organisms living in aquatic habitats, such as fish, amphibians and some marine invertebrates, possessed a particularly large number of Sec residues. Sea urchin SelP with its 28 Sec residues (GenBank: EC436872.1, EC432945.1 and CD311605.1) had a conserved amino-terminal domain, but we could not detect homology of its Sec-rich carboxy-terminal region to other SelPs. The elevated use of Sec in aquatic SelPs might be related to both increased Se utilization and food preferences. Sea urchins mainly feed on algae, which themselves have many selenoproteins [44].

All SelPb sequences had a single Sec, with the exception of *Xenopus *SelPb, which had Cys in place of Sec. Since fish, bird and mammalian SelPb sequences contain Sec, it appears that this residue was replaced with Cys in frog SelPb. PSI-BLAST searches identified a distant Cys-containing homolog of SelP in *Caenorhabditis elegans *(GenBank: NP_494277.2). It contains 23 Cys residues and is annotated as a prion-like-(Q/N-rich)-domain protein. *C. elegans *has only one selenoprotein and requires very little Se. In addition, the lack of Sec in the SelP-like protein precludes its participation in Se delivery in this organism. Therefore, the function of this protein is likely determined by the Trx-fold domain. Another Cys containing homolog was found in the sea anemone, *Nematostella vectensis *(GenBank: XM_001637122).

### Differences between fish and mammalian selenoproteomes

**Figure 3:**
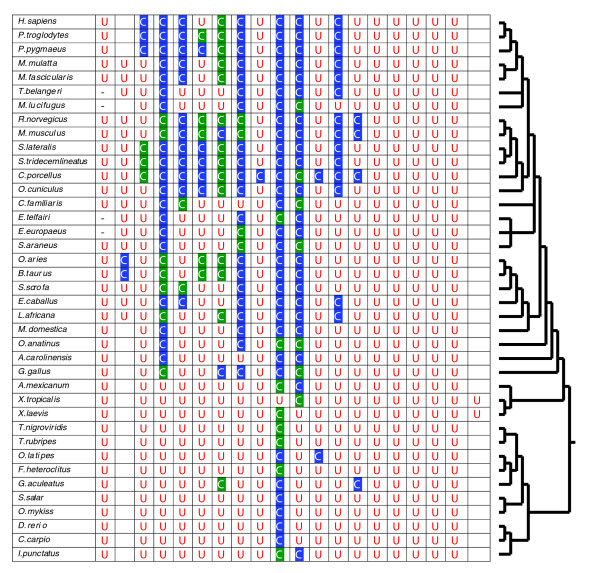
Occurrence of Cys and Sec in vertebrate SelP sequences. The figure shows amino acids that occur in 20 positions, where Sec is found in vertebrate SelPs. Sec residues are shown by the red letter U, and Cys residues by the letter C. Cys residues encoded by TGT are highlighted in blue and those by TGC in green. Hyphens ('-') indicate that sequence information was not available for this region, and empty cells correspond to the situations where an amino acid residue other than Sec or Cys is used.

Comparison of Sec content of SelPs from various organisms revealed that fish contained more Sec residues than mammals (Figure 3; Figures S3 and S4 in Additional data file 1). Mammalian selenoproteomes were previously thought to represent a set of eukaryotic selenoproteins. However, several selenoproteins were identified recently (for example, SelJ, SelL and Fep15) that occur only in fish and several other aquatic organisms. Did these proteins evolve in aquatic organisms after diverging from mammals or were they lost in mammals? More generally, how does the change in Sec content of SelP relate to the changes in the composition of selenoproteomes?

**Figure 4:**
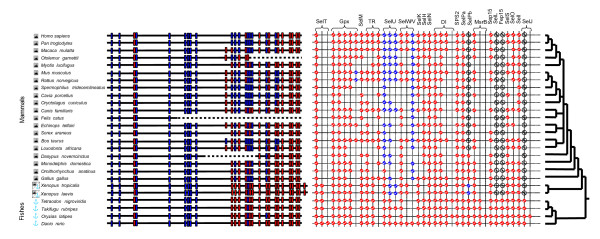
Selenoproteomes and the Sec content of SelPs. Taxonomic tree, Sec and Cys content of SelPs (left panel) and selenoproteomes of vertebrates with completely or partially sequenced genomes (right panel) are shown. Red circles with ticks show the presence of selenoproteins. Blue circles with ticks indicate the presence of Cys-containing homologs. Crossed black circles are used to show the loss of protein during evolution. Similarly, red and blue squares are used to indicate Sec and Cys residues in SelP sequences, respectively. Dotted lines (empty cells) correspond to undetected sequences in genomes with low sequence coverage.

To address these questions, we reconstructed selenoproteomes of all vertebrates for which extensive genome sequence information is available, including 19 mammals, 4 fish, 1 bird and 2 amphibians (Figure 4). Consistent with the high Sec content of fish SelPs, the largest selenoproteomes were detected in these organisms. We recently proposed that aquatic environments may favor increased reliance on Se in lower eukaryotes via unknown environmental factors [44]. Can this aquatic/terrestrial observation be extended to higher eukaryotes? Note that humans and large terrestrial mammals possess the protective cover of skin, which together with their large size may make their intraorganismal environment more similar to that of their aquatic ancestors. Interestingly, comparison of selenoproteomes from aquatic and terrestrial vertebrates revealed an 'aquatic/terrestrial' trend: fish had 32-34 selenoproteins, whereas mammals had 23-25.

However, more important was the observation that several fish selenoproteins had homologs in mammals in which Cys was present in place of Sec (for example, SelU1, SelU2, SelU3, a SelW-like protein Rdx12 and GPx6). In contrast, no fish could be found that had Cys orthologs of mammalian selenoproteins, suggesting unidirectional loss of Sec in mammals. In addition, some fish selenoproteins that also occurred in invertebrates and/or amphibians and birds (for example, SelPb, SelL, Fep15, and SelJ) had no mammalian counterparts, suggesting their loss in mammals. The timing of selenoprotein gene loss and Sec-to-Cys conversions differed for various vertebrate selenoproteins. For example, SelU is a selenoprotein in fish and many lower eukaryotes, and it also occurs in the Sec form in an early mammal, the platypus, whereas other mammals possess only the Cys version. Thus, Sec in SelU was replaced with Cys in early mammals. Likewise, SelPb is found in fish, birds, and frogs, and is present in platypus and opossum, but not in placental mammals (with the notable exception of armadillo, one of the earliest placental mammals). Thus, SelPb also was lost in early mammals, but later than SelU. The other events of selenoprotein loss (for example, of SelL) could be extended to all mammals or to a select group of mammals (for example, GPx6 in rodents).

There are several possible explanations for the decreased content of selenoproteins in terrestrial eukaryotes. First, the loss may be due to lower bioavailability of Se in terrestrial habitats. This would be similar to the decreased utilization of nutrients, such as nitrogen, in certain environments [45] or reduced availability of iron in oxygenated ocean [46,47]. Although the overall concentration of bioavailable Se does not appear to be lower in terrestrial environments [48], aquatic organisms would have the advantage of concentrating this trace element due to constant exposure to the aquatic source of Se. In addition, terrestrial organisms adapted to preserve water; however, this feature might have reduced their exposure to Se and perhaps certain other nutrients and micronutrients in the environment. Whereas the reduced bioavailability of Se would primarily apply to unicellular and small eukaryotes, following the food chain, most terrestrial organisms would reduce their Se content.

A second possibility for the loss of selenoproteins in terrestrial organisms is the extreme reactivity of Sec, which is the same chemical property that makes Se so important to life. Air has higher availability and a higher content of oxygen compared to water, which should make selenoproteins more susceptible to oxidative damage as well as cause damage themselves due to side reactions of Sec. An additional factor for toxicity of Sec may be an increased UV radiation in terrestrial environments, which may result in generation of reactive oxygen species that are capable of damaging selenoproteins. Therefore, the widespread use of these proteins in the face of high oxygen may be detrimental to terrestrial life, although less so for large organisms (this could explain the presence of relatively large selenoproteomes in mammals compared to the selenoproteome size of insects and unicellular organisms). Participation of selenoproteins in essential cellular processes would then pose a serious challenge to organisms that utilize these proteins. The reduced Sec content of mammalian SelPs may then be a consequence of lower Se requirement. Since SelP functions as a Se transport protein, it is possible that organisms with smaller selenoproteomes and lower expression of selenoproteins require less Se.

### Many Sec positions are occupied by Cys in mammalian SelPs

Analysis of the multiple sequence alignment of SelP sequences (Figure S2 in Additional data file 1) revealed strong conservation of Sec residues, although some positions were less conserved than others. The areas of highest conservation included the first Sec and several Sec residues in the very carboxy-terminal region, which were nearly 100% conserved in vertebrates. Interestingly, the majority of less conserved Sec positions were occupied specifically by Cys residues. Sec/Cys pairs in homologous sequences is a characteristic feature of selenoproteins, which accounts for evolution of these proteins [12] and helps identify these proteins in sequence databases [49,50]. Thus, proteins with multiple Sec residues also exhibit this feature, even though their Sec/Cys replacements occur more rapidly than in selenoproteins with a single Sec, which often utilize these residues for catalysis [6].

### Unidirectionality of Sec/Cys transitions in SelP

**Figure 5:**
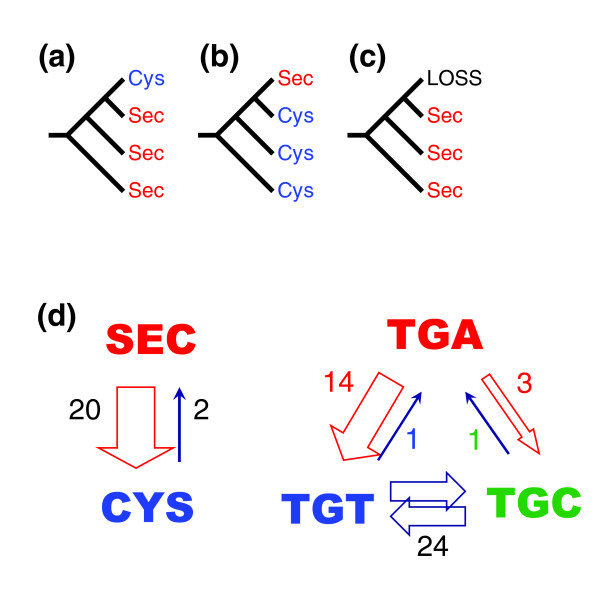
Inference of Sec transitions. **(a) **Sec-to-Cys transition. To infer such a transition in a SelP sequence, we required the presence of two closest outgroup sequences and a sister SelP sequence containing Sec. (**b) **Cys-to-Sec transition. To infer gain of Sec from Cys, we required two outgroup sequences and one sister sequence to contain Cys. **(c) **Loss of Sec. If Sec was present in two outgroup and one sister sequence, whereas the corresponding position in the tested SelP sequence had an amino acid other than Cys or Sec, the inference was Sec loss. **(d) **Sec/Cys transitions viewed at the level of amino acids and codons. Numbers indicate the number of transitions between indicated amino acids or codons. The size of arrows highlights directionality of these transitions.

We analyzed the frequency of Sec-to-Cys and Cys-to-Sec changes in SelP sequences that had the carboxy-terminal Se transport domain (Figure 3). Among 20 positions where Sec residues could be found in at least one vertebrate SelP, 13 positions had Cys forms in some SelPs, indicating that at least two-thirds of Sec residues are replaceable with Cys in SelPs. All 13 Sec/Cys transitions occurred in the Se transport domain of SelP. To quantify Sec/Cys transitions, we considered that if at least two outgroup and one sister sequence had the same amino acid (Sec or Cys), but the other sister sequence had the opposite residue, a Sec/Cys transition could be inferred (Figure 5a,b). Similarly, Sec loss (that is, replacement of Sec with amino acids other than Cys) and origin (replacement of an amino acid other than Cys with Sec) events were quantified (Figure 5c). Due to insufficient information, some Sec/Cys replacements remained unresolved. The use of such strict criteria resulted in some underestimation of Sec/Cys transitions, but provided reliable inferences in both Sec-to-Cys and Cys-to-Sec directions that could then be compared with each other.

With this approach, we detected 20 Sec-to-Cys transitions, but only two Cys-to-Sec transitions. The total number of Cys and Sec residues in analyzed vertebrate SelP sequences was 502 and 418, respectively. Therefore, Sec-to-Cys transitions occurred 12 times more frequently than transitions in the opposite direction. At the same time, the number of Cys-to-Sec transitions was equal to both Sec loss and Sec origin from amino acids other than Cys (that is, two each). Thus, the transitions involving Sec were largely unidirectional and resulted in the replacement of Sec specifically with Cys. As expected, TGA codons for Sec could be replaced in vertebrate SelPs with TGC or TGT (14 and 3 transitions, respectively; 3 additional transitions could not be resolved), and the newly evolved Cys codons had a total of 24 subsequent TGT/TGC transitions (Figure 5d).

### Recent events of Sec loss, switch and gain in closely related species

Analyses of Sec/Cys pairs also identified interesting cases of recent Sec loss and gain events in vertebrates. As shown in Figure S5a in Additional data file 1, even closely related species, such as chimpanzee and human, are characterized by differences in Sec content of SelP; for example, chimpanzee SelP has 9 Sec residues, human 10, and macaque and gorilla 12. Further analysis of SelP sequences indicated that there were recent changes in Sec content in primate SelPs, wherein 2 Sec residues in human and 3 in chimpanzee SelPs were replaced with Cys. Rodent SelPs also had a lower Sec content, with the extreme case being guinea pigs, and all these Sec losses were due to conversion of Sec to Cys. An additional example of the recent change in Sec content is shown in Figure S5b in Additional data file 1, where a Sec in position 354 of SelP was replaced with Cys in *Oryzias latipes*. Interestingly, two *Xenopus *species have 18 Sec residues, but the positions of two Sec in these proteins are different and correspond to Cys in the paired sequence (Figure S5c in Additional data file 1). Combined with the quantitative analysis of Sec/Cys transitions discussed above, the data show that Sec/Cys transitions may go in either direction, or may show an overall neutral transition (which is the situation in frogs) and, therefore, may serve as a sensor of demand for Se.

The analysis of Sec residues in SelPs also allowed us to directly observe the evolution of new Sec residues. Compared to other vertebrate SelPs, *Xenopus *sequences were extended by several residues such that their last Sec codons corresponded to stop signals in fish and mammalian SelPs (Figure S6 in Additional data file 1). We suggest that this example illustrates a mechanism of evolution of a new Sec residue by carboxy-terminal extension, wherein a stop codon (UAA or UAG) changed to a Sec codon (UGA) and the next in-frame stop codon became a new termination signal. A similar mechanism was previously suggested for the evolution of TRs from the glutathione reductase family of proteins [51]. We suggest that the carboxy-terminal domain of SelP evolved *de novo *by extension of its carboxy-terminal sequences.

### SelP expression

Previous studies have shown that mammalian SelP is synthesized primarily in liver [28]. We used UniGene EST ProfileViewer to examine expression levels of SelP in different species *in silico*. Surprisingly, this analysis showed that most ESTs corresponding to *Danio rerio *SelP are derived from kidney. This observation suggests that in *D. rerio *a significant portion of SelP is synthesized in kidney (Figure S7 in Additional data file 1). Liver still appears to contribute significantly to SelP synthesis, but in contrast to mice and rats, to a lower extent than kidney. The number of SelP ESTs in fish was also higher than that in mammals (Figure S7 in Additional data file 1). Thus, not only is the Sec content of fish SelP higher than in mammals, but gene expression of fish SelP is also higher.

### Loss of Sec in mammalian SelPs accounts for differences with fish SelP sequences

Recent, sporadic Sec loss in mammalian SelPs and changes in the composition of selenoproteomes might represent a coordinated response to external pressure, that is, change in habitat that forces an organism to reduce Sec use, which is manifested in both Sec content of SelP and selenoproteomes. Interestingly, the number of Sec residues seems to be inversely proportional to the number of Cys in SelP sequences (Figure S8 in Additional data file 1). Moreover, the number of Sec residues in the most Sec-rich SelPs exceeded that of Cys. If during transit in the circulatory system SelP protects its Sec residues by controlled oxidation, selenenylsulfide bonds may be a preferred chemical form of Sec residues. Indeed, such bonds have previously been observed in rodent SelPs [52]. However, having significantly more Sec than Cys residues, fish and amphibian SelPs are capable of protecting only a fraction of Sec residues through selenenylsulfide bonds. Thus, we predict that Sec-rich SelPs form diselenide bonds that stabilize Sec residues. A disadvantage of diselenide bonds is the difficulty of reducing them because diselenides are characterized by very low redox potentials. Interestingly, we recently identified a protein, SelL, that has a diselenide bond, and the occurrence of this protein is restricted to aquatic organisms, including fish, invertebrates and marine bacteria [53]. Thus, it is possible that diselenide reduction systems occur in aquatic organisms and may act on both SelL and SelP, whereas mammals are unable to reduce diselenides efficiently, lack SelL and utilize selenenylsulfides in SelP.

The relatively frequent replacement of Sec with Cys in the Sec-rich domain of SelP in mammals contrasts with the conservation of Sec in the Trx-fold domain, suggesting that different evolutionary forces act on Sec sites in the amino- and carboxy-terminal domains. This idea is further supported by the occurrence of SelPb (shorter version of SelP) in fish, amphibians and early mammals.

### Should selenoprotein expression be maximized?

The evolved reduced utilization of Sec in mammals raises important questions in human and animal nutrition. Both previous and current clinical trials operate under the assumption that selenoprotein expression should be maximized. Although GPx3 expression is maximized by 55 μg of Se per day (and SelP approximately 100 μg/day), these dietary levels are readily exceeded in the US and most other countries, without dietary supplementation, by consuming regular foods. Clearly, selenoprotein expression is regulated such that humans do not fully utilize the available dietary selenium, any excess of which is excreted in the form of a selenosugar [54]. In this regard, whether selenoprotein expression should be maximized irrespective of health status, genotype, or diet, is not clear, and should be addressed in future studies. The consistent loss of Sec in SelP, replacement of Sec residues with Cys in some proteins, and loss of several selenoproteins in mammals under the conditions when this micronutrient is not limiting suggest a highly regulated and balanced use of this trace element. Selenium is best known for its cancer chemoprevention activity, but previous clinical studies and many studies involving animal models utilized highly contrasting, and often physiologically irrelevant, amounts of Se. It would be particularly important to establish whether Se dietary supplements are useful in situations when disease is not imminent, which is a currently accepted practice. Alternatively, the supplements may be helpful when disruption in redox homeostasis is implicated in disease, or in old age, to alleviate oxidative damage. But it is possible that the supplements should not be used at all and that internal regulation of selenoprotein expression and evolutionary adaptations rather than availability of excess dietary selenium govern the use of this trace element.

## Materials and methods

### Databases and programs

Nucleotide, EST and predicted protein sequences from organisms used in this study were downloaded from NCBI [55]. SECISearch [19] was used for identification of SECIS elements. Stand-alone versions of BLAST and FASTA were used in similarity searches. CLUSTALX was utilized for sequence analysis. Alignment shading was performed using BoxShade web-server [56]. The evolutionary tree was reconstructed using the work of Ciccarelli *et al*. [57]. Missing branches were filled using a maximum parsimony (character-based tree estimation method) approach. The implication was that the preferred phylogenetic tree represents the tree that would require the least number of evolutionary changes. The Protpars program of the PHYLIP package [58] was used to generate a maximum parsimony tree.

### Identification of SelP sequences and homologs of known selenoproteins

SelP sequences were identified with TBLASTN in EST, WGS and NR databases. BLASTN was used to assemble SelP sequences from overlapping sequences. Selenoproteome analysis was carried out using BLASTP, TBLASTN and PSI-BLAST as described elsewhere [49], using a full set of known eukaryotic selenoproteins as a query set of sequences. A specialized version of SECISearch [19] was developed for specific detection of 3'-UTRs of SelP sequences. The modifications included a subroutine for the identification of two SECIS elements located within a single WGS read, EST or other nucleotide sequences. Using this program, we scanned the indicated datasets for sequences containing two SECIS elements on the same strand. A default pattern of SECISearch was used for SECIS element identification. A COVE program [59] with covariance matrix optimized for SECIS elements (AVL and VNG, unpublished) was applied to reduce the number of false positives, and all hits with a COVE score below 15 were dismissed. CLUSTALX was used to prepare multiple alignments.

## Abbreviations

Cys, cysteine; EST, expressed sequence tag; GPx, glutathione peroxidase; RDA, recommended dietary allowance; Se, selenium; Sec, selenocysteine; SECIS, Sec insertion sequence; SelP, Selenoprotein P; TR, thioredoxin reductase; UTR, untranslated region; WGS, whole genome shotgun.

## Authors' contributions

AVL and VNG performed computational analyses. AVL, DLH and VNG wrote the manuscript. All authors read and approved the final manuscript.

## Additional data files

The following additional data are available with the online version of this paper. Additional data file 1 includes supplementary figures S1-S8.

## Supplementary Material

Additional data file 1 Figure S1 shows a search procedure for sequences containing two SECIS elements. Figure S2 features a multiple alignment of vertebrate SelP sequences. Figure S3 shows partial alignment of fish and mammalian SelP sequences. Figure S4 is a plot of selenoproteome size versus Sec content of SelPs. Figure S5 shows recent Sec/Cys changes in SelP sequences. Figure S6 provides an example of evolution of new Sec residues by carboxy-terminal extension. Figure S7 shows an *in silico *expression profile of SelP. Figure S8 is a plot of Cys content versus Sec content of SelPs Click here for file
