# Supplementary material for: Reduced reliance on the trace element selenium during evolution of mammals
Source: Genome Biol. 2008 Mar 31;9(3):R62. doi: 10.1186/gb-2008-9-3-r62 (PMC2397514; doi:10.1186/gb-2008-9-3-r62)
Supplement: Additional data file 1 — Figure S1 shows a search procedure for sequences containing two SECIS elements. Figure S2 features a multiple alignment of vertebrate SelP sequences. Figure S3 shows partial alignment of fish and mammalian SelP sequences. Figure S4 is a plot of selenoproteome size versus Sec content of SelPs. Figure S5 shows recent Sec/Cys changes in SelP sequences. Figure S6 provides an example of evolution of new Sec residues by carboxy-terminal extension. Figure S7 shows an in silico expression profile of SelP. Figure S8 is a plot of Cys content versus Sec content of SelPs [file gb-2008-9-3-r62-S1.pdf]

Additional Data

Figure S1

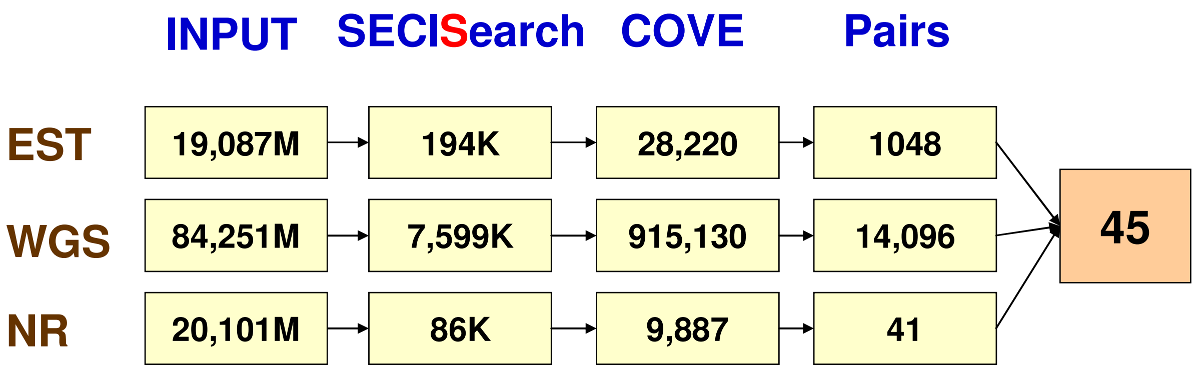

Figure S1. A search for sequences containing two SECIS elements specifically identifies SelP sequences. Statistics of the searches of EST, WGS and NR databases for dual vicinal SECIS elements is shown. Query sequence size ("INPUT") and the number of SECIS candidates in each step ("SECISearch", "COVE", "Pairs") are shown in green boxes. A final number of identified sequences is shown in an orange box, including 43 SelP sequences. Two false positives were also detected in the EST dataset (see text for details).

Figure S2

```
D.rerio                1 -------------MWKALSLTLALCLLVGCSAESETEGARCKLPPEWKVGDV-EPMKNAL
C.carpio               1 -------------MWKALSLTLALCLLVGCSAESETDGARCKLPPVWKIGEE-EPMKNAL
I.punctatus            1 -------------MWRSLSLTLLAALVVGCRGESETDGARCKPAAVWKIGDV-EPLKDSL
T.nigroviridis         1 -------------MRACLGLLLTLCMLHGGGAESDGDGPRCQLPPTWKIGDL-EPMTGAM
T.rubripes             1 -------------MRACLGLLLALCMLHGGGAESEGDGPRCQLPPVWKIGDL-EPMKEAM
G.aculeatus            1 -------------MWAYLSLLVALCLLRGGGAESVGDGPHCQLPSPWRIGEV-EPMQGTM
S.salar                1 -------------MKAGLSLLLALCLLPGGGAESEGEGTRCKPPAGWSIGEV-EPMKGVM
O.mykiss               1 -------MQGLLTLWLCAALPGLLCASPLLVEGDNDASKICKPAPHWEIKGHGAPMKELL
Haplochromis sp.       1 -----MGSLLTLWLFAALPGLLWASHSTLIVEGDNDASRICKPAPQWDIKGY-APMQDLL
O.latipes              1 ----------MLRLCSALPALLWASLSVLSAEGDSNASKICKPAPYWDIEGH-VPMQEHL
F.heteroclitus         1 -----MRSLLVLWLSAALPGLLWVFHSAPPVEGDNDLSRICKPAPNWTIDGS-SPMEERK
X.tropicalis_Cys       1 ------------------MGLLGQVSS-----SEQTNSSICKPPPKWSIEGE-VPMAEAL
X.tropicalis           1 -------------MWKGFALALALCLLPWGGAESQGHRKRCKEPPEWSIGDQ-NPMMQSA
X.laevis               1 -------------MWKGFALALALCLLPWGGAESQGHRSRCKQPPDWSIGDQ-NPMIQSA
A.mexicanum            1 -------------MWGGLGIALVLCILPSGGAESQGPRPHCKKPPDWSIGER-SPMLDSE
H.sapiens              1 -------------MWRSLGLALALCLLPSGGTESQDQSSLCKQPPAWSIRDQ-DPMLNSN
P.troglodytes          1 -------------MWRSLGLALALCLLPSGGTESQDQSSLCKQPPAWSIRDQ-DPMLNSS
P.pygmaeus             1 -------------MWRSLGLALALCLLPLGGTESQDQSSLCKQPPAWSIRDQ-GPMLNSN
M.mulatta              1 -------------MWRSLGLALALCLLPSGGTESQDQSSFCKQPPAWSIRDQ-DPMLDSN
M.fascicularis         1 -------------MWRSLGLALALCLLPSGGTESQDQSSFCKQPPAWSIRDQ-DPMLDSN
E.europaeus            1 ------------------------------------------------------------
T.belangeri            1 ------------------------------------------------------------
L.africana             1 -------------MWRSLGLALALCLLPWGGTESQVQSSFCKQPPAWKIREE-DPMLNSI
E.telfairi             1 ------------------------------------------------------------
M.lucifugus            1 ------------------------------------------------------------
C.familiaris           1 -------------MWRSLGLALALCLLPWGGAESQGQSSFCKQPPAWSIRDQ-NPMLNSS
S.araneus              1 -------------------------------------------------GNE-DPMLNSN
S.lateralis            1 -------------MWR-----------------SQGENFICKQPPDWSIRDQ-NPMQNSH
S.tridecemlineatus     1 -------------MWRSLGLALALCFLPYGGTESQGENFICKQPPDWSIRDQ-NPMQNSH
O.cuniculus            1 -------------MWKSLGLALALCLLPWGGTESHIPSSICKQPPSWSIAEQ-DPMLNSF
O.aries                1 -------------MWRGLGLALALCLLLTGGTESQGQSSYCKQPPAWSIKDQ-DPMLNSY
C.hircus               1 -------------MWRGLGLALALCLLLTGGTESQGQSSYCKQPPAWSIKDQ-DPMLNSY
B.taurus               1 -------------MWRGLGLALALCLLLTGGTESQGQSSYCKQPPPWSIKDQ-DPMLNSY
S.scrofa               1 -------------MWRGLGLALALCLLSWGGTESQGKSSFCEQPPGWSIKDQ-DPMLNSY
R.norvegicus           1 -------------MWRSLGLALALCLLPYGGAESQGQSPACKQAPPWNIGDQ-NPMLNSE
M.musculus             1 -------------MWRSLGLALALCLLPYGGAESQGQSSACYKAPEWYIGDQ-NPMLNSE
C.porcellus            1 -------------MWRSLGLALALCLLPGGGTQSQSKSSYCEQPPPWSIGDQ-NPMQNAT
M.domestica            1 -------------MWRGLGLALALCLLPGGGAESQVQSTRCKDAPKWHIGDQ-DPMQDSL
O.anatinus             1 -------------MWQGLGLALALCLLPGGGAESQSASSHCKEAPRWQIRDQ-DPMLNSL
G.gallus               1 -------------MWAGLGLALVLCLLPGGGTESQR----CQEPPEWHIGEE-SPMLNAR
L.erinacea             1 -------------MQRCLGVALALCLLPVGWAESQG---QCEKPKAWTIGEE-NPMEQSL
S.acanthias            1 -------------MQKGLGVALTLCLLLLGWAESQG---QCEKPKAWTIGEE-NPMEQSL
B.floridae             1 -------RLFLSAGRQSCGPCTTPPVPQETTASPPTETPFCAHPPSWQLDGV-DHLEKSQ
O.porcinus             1 --------------MVRVKIKVASIPLALVASLLLVSQPACGRPPICSQGDR---DLIEG
B.microplus            1 --------------MAARLLVLLA---GCLQATAAARTPICGTPEAWTLGGQ-DPLAAHR
C.gigas                1 ---------------MRSPAGLWLVAWLTAAVLSSALGQTCQRSTLPWRTADGTDPVKMA
A.irradians            1 TSPIKSAYKGGRTIKMRGLGPLLVVWLAAILVASAAGAQRCTRTPIWRTANGTSPMQETE
```

```
D.rerio               47 GQVTVVAYLQASULFCLEQASKLNDLLLKLENQ-GYPNIAYMVVNNREERSQRLHHLLQE
C.carpio              47 GHVTVVAYLQASULFCLEQASKLNDLLLKFENQ-GYVNINYMVVNNRDERSQQLHHLLKE
I.punctatus           47 GRVTVVSFLQASUWFCLSQATRLEELRQKLEDA-GFVNITYMVVNSQDENSLRVHSLLKK
T.nigroviridis        47 GRVTVVALLDASULFCLVQASRMDSLRQKLENQ-GLRDVVYMVVNHQGAQARGLHAMLAQ
T.rubripes            47 GRVTVVALLESSULFCVVQASRMDSLRQRLENQ-GLRDVVYMVVSHQGAHAPGLHAMLAQ
G.aculeatus           47 GRVTVVALLQASULFCLVQASRMDGLRQKLESQ-GLKDVAYMVVNQQGEQARRLHPMLAQ
S.salar               47 GQVTVVALLQASULFCLVQASLLDELRLKLEGQ-GLDNVTYMVVNHQGDQAQHLHTLLSQ
O.mykiss              54 GNVVVLALLKASUHFCRTQASKLGGLRDKLLRS-NLTDVSFLIVNEREAQSRAMYWELKR
Haplochromis sp.      55 GNVAVVALLKAAUQFCLRQASKIGGLRDRLNRS-NMTEVSFIIVNERDAHSRAMYWELKR
O.latipes             50 GNVVVVALLKATUEFCLTQASKIGNLRDKLNRN-NITEVSFMIVNELEALSQTMHWKLKK
F.heteroclitus        55 GNVVVVALLKASUELCLSQASRIGRLRTKLTSR-NITGVSFVIVNDQDAVSRSKYKELKK
X.tropicalis_Cys      37 GKVTVVALLQASCGFCLVQAARMGPLRYKLSLQ-GMTDIKYMIVNDQSLHSANMFPELKR
X.tropicalis          47 GKVTVVALLQASUSLCLLQASRLEDLRLKLEKE-KLVGISYVVVNHQGRQSRAKYDLLKS
X.laevis              47 GKVTVVALLQASUYLCLLQASRLEDLRLKLEKE-KLVGISYVVVNHQGRHSRAKYDLLKS
A.mexicanum           47 GQVTVVALLEASUYVCLLQASRLEELRSKLEKD-GLVNVSIVVVNHQGKASQQAYHLLKE
H.sapiens             47 GSVTVVALLQASUYLCIIEASKLEDLRVKLKKE-GYSNISYIVVNHQGISSRLKYTHLKN
P.troglodytes         47 GSVTVVALLQASUYLCILQASKLEDLRVKLKKE-GYSNISYIVVNHQGISSRLKYTHLKN
P.pygmaeus            47 GSVTVVALLQASUYLCILQASKLEDLRVKLKKE-GYSNISHIVVNHQGISSRLKYTHLKN
M.mulatta             47 GSVTVVALLQASUYLCILQASKLEELRVKLEKE-GYSNISYIVVNHQGISSRLKYTHLKN
M.fascicularis        47 GSVTVVALLQASUYLCILQASKLEELRVKLEKE-GYSNISYIVVNHQGISSRLKYTHLKN
E.europaeus            1 ------------------------------------------------------------
T.belangeri            1 ------------------------------------------------------------
L.africana            47 GSVTVVALLQASUYLCILQASRLEDLRVKLEKE-GYSNVSYVIVNHQDLNSRLKYIHLKN
E.telfairi             1 ---------------------RLENLRVKLEKE-GYSNISYVIVNHQSLVSQLKYTHLKD
M.lucifugus            1 -------------FVCIFPKYFSLEDLRVKLEKEGFSNISYVVVNHQGHPSRLRYNLLKN
C.familiaris          47 GSVTVVALLQASUYLCILQASRLEDLRVKLEKE-GFLNISYVVVNHQGLSSQLKYMYLKN
S.araneus             11 GSVTVVALLQASUHLCIIQASTLEDLRVKLEEE-GYSNISYVIVNHQGFHSQLRYRHLKN
S.lateralis           30 GSVTVVALLQASUYLCILQASRLEDLRIKLEKE-GYSNISYIVVNHQGISSQLKYIHLKN
S.tridecemlineatus    47 GSVTVVALLQASUYLCILQASRLEDLRIKLEKE-GYSNISYIVVNHQGISSQLNYIHLKN
O.cuniculus           47 GSVTVVALLQASUYLCILQASRLEELRVKLEKE-GYSNISYVVINHQGIDSQLKYIHLKN
O.aries               47 GSVTVVALLQASUYLCILQASRLEDLRVKLEKE-GYSNISYVVVNHQEISSRLKYAHLKN
C.hircus              47 GSVTVVALLQASUYLCILQASRLEDLRVKLEKE-GYSNISYVVVNHQEISSRLKYVHLKN
B.taurus              47 GSVTVVALLQASUYLCILQASRLEDLRVKLEKE-GYSNISYVVVNHQGISSRLKYVHLKN
S.scrofa              47 GSVTVVALLQASUYLCILQASRLEDLRVKLEKE-GYSNISYIVVNHQGIASQLKYVYLKS
R.norvegicus          47 GTVTVVALLQASUYLCLLQASRLEDLRIKLENQ-GYFNISYIVVNHQGSPSQLKHAHLKK
M.musculus            47 GKVTVVALLQASUYLCLLQASRLEDLRIKLESQ-GYFNISYIVVNHQGSPSQLKHSHLKK
C.porcellus           47 GTVTVVALLDASUYVCILQASR--------------------------------------
M.domestica           47 GRVTVVALLQASUYLCILQASRLEDLRMKLERE-GFSNISYVVVNHQGNASRLNIKELQD
O.anatinus            47 GTVTVVALLQASUYLCILQASRLEDLRVKLENE-GYSNISYIIVNHQGMPSQLNHKTLKE
G.gallus              43 GSVAVVALLQASUYLCLLQASRLEDLRVKLENE-GLVNISYVVVNHQSPHSQKKFHLLQE
L.erinacea            44 GRVTVVSLLQASUHFCLVQAASLKRLKHSLDHR-GFVNISYLVVNHQANDSRRNYQQLKE
S.acanthias           44 GRVTVVSLLQASUHFCLVQAASLMKLQ-QKLDQAGLVNISFMVVNHQGKRSRGKYQLLKS
B.floridae            53 GQVAVLQFVFASURFCRSQAEKFESLRSTLVEG--LTDISFGAVNGHLYNAPLEIGEIEG
O.porcinus            44 GNVTLVALLSASURLCWRQATGLESLLQDLRLQHGMHKLQFVIVNSMAPNDTVNVDQLSS
B.microplus           35 GNVTLVALLKASUSLCRQQASGLEALLQRLRPN--MTELQFLVVNDRKSAH------LRD
C.gigas               46 GNVTVVALLLASUPMCQQQAEGLEKLMTSYQNI-GKRDISFFIVNHARGEDSVN--ELTR
A.irradians           61 GKVRLVALLKASUGFCVRVAMALERIRSSFERR-GLTGYKFMIVNMNLADAIANVKNLQD
```

```
D.rerio             106 RLLN-ITLYAQDL--SQPDAWQAVNAEKDDILVYDR-CGRLTYHLSLPYTIL------SH
C.carpio            106 RLMN-ITLYAQDL--SQPDVWQAVNVEKDDILVYDR-CGRLTYHLSLPYTIL------SH
I.punctatus         106 KLSDNITLYKQNP--EEPNVWSMAKAEKDDFQIYDR-CGRLTHHLSMPYTIL------SQ
T.nigroviridis      106 RLSEHISLHRQDE--ALADVWQTLGGNKDDFFIYDR-CGRLTHRISLPYAVI------GH
T.rubripes          106 KLTEHISLYKQDE--ALPDVWQTLGGNNNDFFIYDR-CGRLTHRISLPYSII------GH
G.aculeatus         106 RLSVNIDLYKQDE--QQPDVWKTLGGDKDDFLVYDR-CGRLTHHIALPYSII------GQ
S.salar             106 KLSENIILYKQEP--KQADVWQALAGKKDDFLIYDR-CGRLTHHIFLPFSIL------GT
O.mykiss            113 RAPPGIPVYQQAP--LQDDVWEALDGDKDDFLVYDR-CGRLTFHIVLPYSFL------HY
Haplochromis sp.    114 SAPTGVPVFQQQP--LQNDVWEALDGDKDDFLVYDR-CGLLTFHIVMPYSFL------HH
O.latipes           109 KAPTGVPVYQQSS--LQKDVWEILDGDKDDFLIYDR-CGLLTFHIVLPNSFL------QN
F.heteroclitus      114 RAPERVPVYQQSA--NQSDVWELLEGDKDDILIYDW-CGHLTFHMVLPYTLL------HN
X.tropicalis_Cys     96 WAPEGIPVYQQTP--GQDDVWELLDGNKDDFLIYDR-CGRLTFHVRLPLSFL------HF
X.tropicalis        106 KVSEHIPVYQQEE--YQPDVWSLLKGDKDDFFVYDR-CGRLVQHLELPYSLL------HF
X.laevis            106 KVSEHIPVYQQEE--NQPDVWSLLKGDKDDFFIYDR-CGRLVQHLELPYSLL------HF
A.mexicanum         106 KVSDHIPVYQQDE--GQPDVWNTLNATKSDFLIYDR-CGRLVSHLGLPYTFL------SF
H.sapiens           106 KVSEHIPVYQQEE--NQTDVWTLLNGSKDDFLIYDR-CGRLVYHLGLPFSFL------TF
P.troglodytes       106 KVSEHIPVYQQEE--NQTDVWTLLNGSKDDFLIYDR-CGRLVYHLGLPFSFL------TF
P.pygmaeus          106 KVSEHIPVYQQEE--NQTDVWTLLNGSKDDFLIYDR-CGRLVYHLGLPFSFL------TF
M.mulatta           106 KVSEHIPVYQQEE--NQTDVWTLLNGSKDDFLIYDR-CGLLVYHLGLPFSFL------TL
M.fascicularis      106 KVSEHIPVYQQEE--NQTDVWTLLNGSKDDFLIYDR-CGLLVYHLGLPFSFL------TL
E.europaeus           1 -----------------------------------R-CGRLVYHIGLPFSFL------SF
T.belangeri           1 --------------------------------LFSR-CGRLVSHLGLPFSFL------TF
L.africana          106 KVSEHIPVYQQEE--NQTDVWTLLNGNKDDFLIYDR-CGRLVYHLGLPYSFL------TF
E.telfairi           39 QVSENITVYQQEE--NQTDVWTLLDGSKDDFLIYDR-CGRLVYHLGVPYSFL------TS
M.lucifugus          48 KVSEHIPVYQQEE--NQTDVWTLLNGNKDDFLIYDR-CGRLVYHLGLPFSIL------EF
C.familiaris        106 KVSEHIPVYQQEE--NQTDVWTLLNGKKDDFLIYDR-CGRLVYHLGLPYSFL------TF
S.araneus            70 QVSEHIPVYQQEE--NQTDIWTLLNGNKDDFLVYDRYCKYVCTVENNFKFI-=-----YE
S.lateralis          89 KVSDHIPVYQQEE--NQTDVWTLLNGYKDDFLIYDR-CGHLVYHLGLPYSFL------TF
S.tridecemlineatus  106 KVSDHIPVYQQEE--NQTDVWTLLNGYKDDFLIYDRS-----------------------
O.cuniculus         106 KVSHHILVYQQEE--NQTDVWTLLKGSKDDFLIYDR-CGRLVYHLALPYSFL------TF
O.aries             106 KVSEHIPVYQQEE--DQPDVWTLLNGNKDDFLIYDR-CGRLVYHLGLPYSFL------TF
C.hircus            106 KVSEHIPVYQQED--DQPDVWTLLNGNKDDFLIYDR-CGRLVYHLGLPYSFL------TF
B.taurus            106 KVSEHIPVYQQEE--NQPDVWTLLNGNKDDFLIYDR-CGRLVYHLGLPYSFL------TF
S.scrofa            106 KVSEHIPVYQQEE--NQTDVWTLLNGNKDDFLIYDR-CGLLVYHLGLPYSFL------TF
R.norvegicus        106 QVSDHIAVYRQDE--HQTDVWTLLNGNKDDFLIYDR-CGRLVYHLGLPYSFL------TF
M.musculus          106 QVSEHIAVYRQEE--DGIDVWTLLNGNKDDFLIYDR-CGRLVYHLGLPYSFL------TF
C.porcellus          69 -----ITLY----------LYPLL-------LPFFR-CGRLAYHLRMPYSFL------SF
M.domestica         106 QVSKNITVYQQEQ--NQKDVWTTLNGNKDDFLIYDR-CGRLVYHLGLPHTFL------SF
O.anatinus          106 KVSEHIPVYQQDE--KQTDVWSTLKGNKDDFLIYDR-CGRLVYHLSLPYTFL------SF
G.gallus            102 SVSDHITVYQQDD--HQADVWTTLNGNKDDFLIYDR-CGRLVYHLGLPYSFL------SF
L.erinacea          103 KVEGQLPVYQQGT--DQPDVWNLLRGEKDDFLIYDR-CGQQTFHLGLPFTIL------SQ
S.acanthias         103 KVTEEIPVYQQDS--NKPDVWELLQGDKDDFLIYDS-CGKLTHHLQLPFTIL------TD
B.floridae          111 RVGFPVYQDT-----AEEEIWKLLDARKDDFIIYDR-CGRLVRHIRMPQANM------DN
O.porcinus          104 RATFPVLQETAEK-----PIWRELHGEKGDFFLYDR-CGRLVYFLPFPLTKFPE---LMS
B.microplus          95 HLEQRVSFAVHQETA-EEPVWTTMEGGKDDVFIFDR-CGRLAFYVPFPISLVLP---GHL
C.gigas             103 RVSFPVYQDDNTS-----MIQQTLNASIDDLFLYDR-CGTLVYHLRKPESLV------SH
A.irradians         120 VVK-----DDQLE------IWRKLGGDKDYLYFYDG-CGMFRFLLTSDDILDREQNSINT
```

```
D.rerio              156 PHVEEAIKHTYCDRICG-ECSLESSAQLEECKKATEEVNKPVEEEPRQDHGHHE------
C.carpio             156 PHV---------------------------------------------------------
I.punctatus          157 PHVEEAIRNAYCAAVCG-ECELERSDQLEECNKTKEEKTEETPKTEEEDHHHHQ------
T.nigroviridis       157 GHVEKAVKDTYCSSLCG-ECTHETTETQQECTPKTDTQPQ-------EDTRHEC------
T.rubripes           157 GHVERAVKDTYCNSLCG-ECTHETTETLQECTPKTSALPDNGVAPGAEETGHEC------
G.aculeatus          157 GHVESAIKDAYCKRTCG-DCVHESATTPEECVEKAVAQPDADAPPVVEDNGGGG------
S.salar              157 PYVENAIKETYCQSICG-DCTYESTEIPAECNRMVEVKPEGEEKP---VTGGDT------
O.mykiss             164 PYIEAAVRATYHKDICG-NCTVDSTQPPQ-------------------------------
Haplochromis sp.     165 PFVEAAIRATYQKNIC--NCTHNSTSS---------------------------------
O.latipes            160 ADVENAITATYTQDIC--NCSGNST------LSGGGNNFTRNSSQSHSG---SPAGEEHQ
F.heteroclitus       165 THVENAISATYQGKHLQ-------------------LLHRDKPRRGYRTIVI------GH
X.tropicalis_Cys     147 PYVEAAILSTYNESFCG-NCSFTSNST---------------L-----------------
X.tropicalis         157 PYVEEAIRIAYCEDKCG-ECEHKILDAD-VCKKPEEQQEQDKPVEEKVERPRHHR-----
X.laevis             157 PYVEEAVRIAYCGDKCG-ECEHKIPDAD-VCKKPEEQPEQEKPVEEKVERPRPHR-----
A.mexicanum          157 PYVGESIRSAYCENKCG ICKNTIESVAEVCNASLKLPAEGVVEEQQPEDTPKTHRHEPR
H.sapiens            157 PYVEEAIKIAYCEKKCG-NCSLTTLKDEDFCKRVSLATVDKTVETPSPHYHHEH------
P.troglodytes        157 PYVEEAIKIAYCEKKCG-NCSLTTLKDEDFCKRVSLATVDKTVETPSPHYHHEH------
P.pygmaeus           157 PYVEEAVKIAYCEKKCG-YCSLTTLKDEDFCKSVSLATVDKTVEAPSPHYHHEH------
M.mulatta            157 PYVEEAIKIVYCEKKCG-NCSLTTLKDEDFCKSVSLATVDETVEAPQPHYHHEH------
M.fascicularis       157 PYVEEAIKIVYCEKKCG-NCSLTTLKDEDFCKSVSLATVDETVEAPQPHYHHEH------
E.europaeus           19 PYVEEAIKITYCEKTCG-NCSHTTLENGGFCKNISLATSENTTIA--PKHHHHD------
T.belangeri           22 SYVEEAIKSAYCEEKCG-NCSLT-------------------------------------
L.africana           157 PYVEAAIKIVYCEEKCG-NCSLMTPEDEDFCKKVSSVNVEDTAEASKPHHHHSH------
E.telfairi            90 PFVEAAIKTAYCEKKCG-NCSLMNLEDEAVCKGIASASVQEATEAAKPHHHHHH------
M.lucifugus           99 PYVEQAIKIAYCEEKCG-NCSLT-------------------------------------
C.familiaris         157 PYVEEAIKRAYCEEKCG-NCSLTVLEDEEVCKMVSSGTVESTTEAPQPHPHDHH------
S.araneus            121 R-LLYLLQTLKHEEFCK-NISLASAEKPTEAPHHFHHQPH--------------------
S.lateralis          140 PYVEDAIKIAYCEDRCG-NCSFRTLENEDFCRNVSFPTVEKTIEALQPHHHHN-------
S.tridecemlineatus   141 ---------------------LQTLENEDLCRNVSLLTVEKTTEALQPHHHHN-------
O.cuniculus          157 PYVEEAIKIAYCEETCG-NCSLMTPEDESFCKNVSLATVEKVSEALRPHHHHTH------
O.aries              157 TYVEDSIKTVYCEDKCG-NCSLKTQEDEDFCKNVSLATRRKQLRL---------------
C.hircus             157 TYVEDSIKTVYCEDKCG-NCSLKTQEDEDFCKNVSLATKEK-------------------
B.taurus             157 TYVEDSIKTVYCEDKCG-NCSLSRPQDEDVCKNVFLATKEKTAEASQRHHHPHP------
S.scrofa             157 PYVEEAIKTVYCENKCG-NCSLKTLKDEDFCKNVYLATEEKTTEAPQPHHHHDH------
R.norvegicus         157 PYVEEAIKIAYCEKRCG-NCSFTSLEDEAFCKNVSSATASKTTEPSEEHNHHKH------
M.musculus           157 PYVEEAIKIAYCEERCG-NCNLTSLEDEDFCKTVTSATANKTAEPSEAHSHHKH------
C.porcellus          100 PYVEQAIKIVYCVEKCG-NCSLKNLEDDDFCKNVS-LAAVTTEAPQKHHHRQIH------
M.domestica          157 SYVEDAIKSAYCEKACG-NCSYTTLDDEGFCKNVSLVAEEETITTSLHHHYHPP------
O.anatinus           157 SYVEDSIKTTYCEQNCG-NCSYTMPEAEEFCTNTSSAAKEKATEAPLPHNDRPH------
G.gallus             153 QYVEEAIKIAYCENNCG-NCSYTEPDIDNICENITLAGIEPEPEPSGQHSHHHH------
L.erinacea           154 VYVEEAIVQTYCHTMCT-NCSL-VEVPLE-CLAR--------------------------
S.acanthias          154 LYVEYAIRQTYCQEICA-NCSL-VHDPPA-CSAR--------------------------
B.floridae           159 SDVEDAIRAVYEESPCG-PCTTP-------------------------------------
O.porcinus           155 SVTRKVLLETYYRSLCGNACAPN-------------------------------------
B.microplus          142 PVVEDSLRRAYDGEPCGLTCEEPTE-----------------------------------
C.gigas              151 GTMQTNLLTTYLGNLCK--CTEKFKS----------------------------------
A.irradians          168 RLIRRRLRQVSRRDRCARQCRR--------------------------------------
```

```
D.rerio               209 --HG--------------------HHEHQGEAERH------RHGHHHPHHHH--------
C.carpio              159 ------------------------------------------------------------
I.punctatus           210 --HH--------------------HHHGHHEGHHH------RGHHHGHHPHDGVETRGGS
T.nigroviridis        203 --HHRHH-----------------QGHQHHGDGHR------DHGDNQCTHTQGSGPGHGH
T.rubripes            210 --HH--------------------HGRQHHGDGHR------DHGDSQCTHTRGSGRGHGH
G.aculeatus           210 --GH--------------------HGHHHHGHGH--------HRGHHHGHHHG--HHHGG
S.salar               207 --PHGG------------------RGHHHHGNGHG------HHSKSHGHGHHGE-SEVGR
O.mykiss              192 ------------------------------------------------------------
Haplochromis sp.          ------------------------------------------------------------
O.latipes             209 HQHPHHGHHGHNHGDNHGLHPR-----------------------GFGHGHDHRHGHHHR
F.heteroclitus        200 I-H---------------------------------------------------------
X.tropicalis_Cys      174 --IPMN------------------------------------------------------
X.tropicalis          210 --NHHR-----------------HHRPKHSGHR---------HRHHHSEDGQVAELDVLR
X.laevis              210 --NHHR-----------------HHRPKHSGHR---------HRHHNNEGGQAAEVDAF-
A.mexicanum           188 -GHGHR--HHHHQQH---NHER-DHLPEDPDSKP--------HPHHHNR-----------
H.sapiens             210 --HHN-------------------HGHQHLGSS---------ELSENQQPGAPNAPT-HP
P.troglodytes         210 --HHN-------------------HGHQHLGSS---------ELSENQQPGAPNAPT-HP
P.pygmaeus            210 --HHN-------------------HRHQHLGSS---------ELSENQQPGAPDAPT-HP
M.mulatta             210 --HHN-------------------QGHQHLGSS---------ELSENQQPGAPDAPT-HP
M.fascicularis        210 --HHN-------------------QGHQHLGSS---------ELSENQQPGAPGAPT-HP
E.europaeus            70 --HHG-------------------LGHQHAGNG---------PLSENQQPGAQGAPE-HF
T.belangeri            44 -------------------------GISMLGEI---------SFQENQQPGTHDDPL-HL
L.africana            210 --HHS-------------------HGHQHLGNH---------QHSENQQPGDPEAPQ-HS
E.telfairi            143 --HHH-------------------HHHHRPGHQH-----ENSQPSENQSPSEPDTIQ-HP
M.lucifugus           121 ------------------------------------------------------------
C.familiaris          210 --LHH-------------------HHHHHHKHWHRLMPHGNDELSENQQPEEPDVSE-HP
S.araneus             159 --HHR-------------------HGHQQLGHP---------QFSEHQQPEKTEASE-HP
S.lateralis           192 --------------------------HQHLGSS---------ELSKNQQPGAADTPS-NP
S.tridecemlineatus    173 --------------------------HQHLGSS---------ELSENQQPGAADTPS-NP
O.cuniculus           210 --HHK-------------------HGHQHLDSS---------ELSEKQHPGTPDAPL-HL
O.aries               201 -----------------------HSDITIT------------WTPAGTGPEFPGRPT-RP
C.hircus              197 ----------------------------------------------------TAEAS-QR
B.taurus              210 --HSH------------------HHGHQLHENA---------HLSESPKPDTPDTPE-NP
S.scrofa              210 --HHHRHHH-------------HHHGHQHLGNG---------HLSEHPKPEAPDTPE-QP
R.norvegicus          210 --HDK-------------------HGHEHLGSS---------KPSENQQPGALDVET-SL
M.musculus            210 --HNK-------------------HGQEHLGSS---------KPSENQQPGPS--ET-TL
C.porcellus           152 --PPK-------------------RGHPYLGSR---------EPEAPDNPVQS-------
M.domestica           210 --HRHG-----------------HHPHHHHGHHHPPPLG--HPPSENDKPEGSEGAV-HS
O.anatinus            210 --HHH-------------------HHHHHHGHKP-------HPSGTEQAPADPDGPLRSP
G.gallus              206 --QLHR------------------HRHHHHHREG-------GRHSKNQNHQAPSESQRRH
L.erinacea            185 ------------------- ----------------------------------------
S.acanthias           185 ------------------- ----------------------------------------
B.floridae            181 ------------------------------------------------------------
O.porcinus            178 ------------------------------------------------------------
B.microplus           175 ------------------------------------------------------------
C.gigas               175 ------------------------------------------------------------
A.irradians           190 ------------------------------------------------------------
```

```
D.rerio              233 --------HHHRGQQQVDVDQQVLSQVDFGQVAVETPMMKRP--UAKHS-RUKVQYS-UQ
C.carpio             159 ----------------------MLAQVDFGQAAIEPPVMKRP--UAKHT-RUKVQYS-UQ
I.punctatus          242 NQQHGHEGQVQVQRSQVDLGQAHVGQIDLGQVGINQQVMRRP--UAS---RUKFQFM-CQ
T.nigroviridis       238 GH-HGGQGH-HQGHDQAGGVAQRPDHLELGQAQ-HDAAAAKP--UESK--RUKF-QFSUQ
T.rubripes           242 HHGHGGQGQ-HEGRVHMGDIPQRPDHLDLGQAQQHDAAATRP--UESK--RUKA-QFSUQ
G.aculeatus          238 DHGAGQQAVVHQEHERDGGASHGQHNSALDQMQQAQAAPVRP--UVEENAKUKS-KHSUK
S.salar              240 DHGRGHGVEQQQHQHGAEGLHHGQAHGQLHVGQEEGHIMQRP--UVKGRARUKA-ELSUH
O.mykiss             192 -----------------------------------------------------VEGGVUS
Haplochromis sp.         ------------------------------------------------------------
O.latipes            246 HHHGRAETRLQEHQHHA--SSDQMQHAVQLEQIGQEVVGAPVRPUVQETARUKTK-FTUH
F.heteroclitus       202 ---------------------GIGRRSVDTQQFSQEVLAFPVRPUAADNVRUKSK-FTUQ
X.tropicalis_Cys     178 ------------------------------------GTTVSP-----------------S
X.tropicalis         242 SSAQANNRAGSQNGQGQSQVVPQSEVLF-VPQREADIPVLARQPUKKA-KSUKK-QYLUD
X.laevis             241 ---QTNNRAGSHNGQGQS-VVPQSEVVF-VPQREADVPVLALQPUKKA-KSUKK-QYLUE
A.mexicanum          210 --AMGPNRHGQRSG-QQVVDALPQPDVVGGSPQREMKRPNRIRQUNK--QSUNK-QFMUD
H.sapiens            239 APPGLHHHHK---HKGQHRQGHPENRDMPA-SED--LQDLQKKLCRK---RCIN-QLLCK
P.troglodytes        239 APPGLHHHHK---HKGQHRQGHPENQDMPG-SED--LQDLQKKLCRK---RCIN-QLLCK
P.pygmaeus           239 APPGLHHHHK---HKGQHRQGHPENRDMPG-SED--IQDLQKKLCRK---RCIN-QLLCK
M.mulatta            239 APPGLHHHHK---HKGQHRQGHPESUDMPG-SEG--LQHLQKKLURK---RCINPQLLCK
M.fascicularis       239 APPGLHHHHK---HKGQHRQGHPESUDMPG-SEG--LQHLQKKLURK---RCIN-QLLCK
E.europaeus           99 APLGHRHPHR---HKGSHGEGHPEDUDMAG-SGRLHLSLPQKRLURK---GCIN-QLLUK
T.belangeri           69 PHTGLHHHHK---HKGHDRQGHPESUDMP--------ELLQKKIURK---GCIN-QLLUQ
L.africana           239 PPPGLHHHHK---HKGPQRQGHPESUDMPG-SEGLQPSLPQKKLURK---GCIN-QLLUQ
E.telfairi           176 SPLDLHRHYK---HKGHESQGHPGNUDMPG-SESLQLSLPRKQLURK---RCRN-QLLUK
M.lucifugus          121 -----------------------------------PLSSPQKKLUGK---RCLN-QLLUK
C.familiaris         248 APQGLHRHHK---HKDHQRQGHPDNUDMPAGSESLQLSVPQNQLURK---GCRN-QLLCK
S.araneus            188 SLTHLHHHHK---HKGPPSQGDPENUDMPA-SGGSQLSLPQKKLURK---RCIN-QLLUK
S.lateralis          216 APLDLHHHKK---HKGQYRQGNSESUQMAG-SEGLQLSLAQRKLCQK---GCVN-QLQCK
S.tridecemlineatus   197 APLDLHHHKK---HKGQYRQGNSESUQMAG-SEGLQLSLAQRKLCQK---GCVN-QLQCK
O.cuniculus          239 PPSGLHHHHK---HRDQHRQGNQESUDMPE-SESLQLSLVRKKLUQR---GCLN-QLRCK
O.aries              225 -----HHHHHR--HKGHQRQGHSDNCDTPVGSENLQLSLPQKKLURK---RCIN-QLLUQ
C.hircus             204 -----HH-----------------------------------------------------
B.taurus             240 PTSGLHHHHHR--HKGPQRQGHSDNCDTPLGSESLQPSLPQKKLURK---RCIN-QLLUQ
S.scrofa             245 PPSGLHHHHG---HKGHQRQGHSENUDMPAGSESLQLSLPQKKLURR---GCIS-QLLCK
R.norvegicus         239 PPSGLHHHHHHHKHKGQHRQGHLESUDMGA-SEGLQLSLAQRKLURR---GCIN-QLLCK
M.musculus           237 PPSGLHHHHR---HRGQHRQGHLESUDTTA-SEGLHLSLAQRKLURR---GCIN-QLLCK
C.porcellus          175 LPLGLHNLHR-----GQPRQVHSPSUDMVP-GEGFQPSPQLRKLCPK---GCKT-QVLCK
M.domestica          248 HPTQGLHHHHEAAGPQHRHTDHPESQENPEISVS-ELSVPRKKLURKGISGCAN-QLLUN
O.anatinus           242 APQGLHKRLR---PAGQPRQGQGGSREAAE-GRGEELPSPRKKAURKGNASCQN-QLLUD
G.gallus             239 PHNGRRHRVFN--HNRHDQIGSHEQVETLPPGEGVENLPRVTKLUKKGKTICKN-QLTUN
L.erinacea           185 ---------------------NATQPKVVG--GHRGKHNPHR------------------
S.acanthias          185 ---------------------TEDIKT-----EEDDLHSHRNHN----------------
B.floridae           181 ------------------------------------------------------------
O.porcinus           178 ------------------------------------------------------------
B.microplus          175 ------------------------------------------------------------
C.gigas              175 --------------------------------------------------KVSTKPTLYQ
A.irradians          190 ---------------YQSRLMSAADMDLMQGHQPNVVSMPVGQSSSSDDNRTPACMELHC
```

```
D.rerio               281 QGA--DSPVA---SUCUHURQLFGGEGNGRVAGLUHCD-EPLPASUPUQGLKEQ-DNHIK
C.carpio              193 QGA--DASAT---SUCUHURQLFD-DSNGHVAGLUHCE-GALPASURUQGLKE--DNHIR
I.punctatus           296 QGA--LSDPS---SUCUHURRLIGLHLNERP--LUQCD-EPLAASCLUQGLLTD-QNNM-
T.nigroviridis        290 WTEASDPDASPKASUCUHURRLFGSVGSERPAGLURCS-EALPASUQUHGPMADRADA-R
T.rubripes            296 WAEASDTGAFPKASUCUHURRLFGDVVGEEPVGLUHCS-EALPASUQURGPTGDAVNAVR
G.aculeatus           295 LTAGSDNEASLKLSUCCHURRLFGEVGSEQPLGLUHCD-EALPASUQUHGLTDGVPNNVR
S.salar               297 LKEGSDISPSSKVSUCUHURGLFGNGVSNEPIGLUHCD-EALPASUQUQGLMGDSTNHIR
O.mykiss              199 KS---LFQ----ASUCUHURRLFGDGVSNEPIGLUHCD-EALPASUQUQGLIG-----LR
Haplochromis sp.          ------------------------------------------------------------
O.latipes             303 MVAGSENE----ASUCUHURRLFGHAGSEQPLGLUHCQ-EELPASUQCRGLTGELANTVI
F.heteroclitus        240 GAAGSDNEAAPKAGUCUHURRMFGEAADEQALGLUHCE-GALPASUQUQGLTSEAAAAVK
X.tropicalis_Cys      185 GD---DSSSP--------------LQNKDEPVNK-EPS-PTLEKHNDQRKLDSELR--LH
X.tropicalis          299 WR---EEGGKAFNSUCUHURQLSFEVTENEVA--UHUQ-EALPSSCSUQELLS---DSLP
X.laevis              294 WR---EDAGKAFNSUCUHURQLSFEIAQNEVA--URCQ-EALPASUQUQELLS---DSLS
A.mexicanum           264 QQ---DVSGSASSSUCUHURQLSLSESPKAVT--UQCN-SVLPSSCKUQEQLS---EGAS
H.sapiens             289 LP---TDSELAPRSUCCHCRHLIFEKTGSAIT--UQCK-ENLPSLCSUQGLRAE--ENIT
P.troglodytes         289 LP---KDSELAPRSCCCHCRHLIFEKTGSAIT--UQCK-ENLPSLCSUQGLRAE--ENIT
P.pygmaeus            289 LP---KDSELAPRSCCCHCRHLIFEKTGSAIT--UQCK-ENLPSLCSUQGLRAE--ENIT
M.mulatta             290 LP---KDSELAPRSUCCHCRHLIFEKTGSAIT--UQCK-ENLPSLCSUQGLLAE--ENIT
M.fascicularis        289 LP---KDSELAPRSUCCHCRHLIFEKTGSAIT--UQCK-ENLPSLCSUQGLLAE--ENIT
E.europaeus           151 LP---KESELAPSNUCUHCRHLVFEKTGSVTT--UQCS-ENLPSLCSUQGLWAA--ENVI
T.belangeri           114 LP---RDSELAPRSUCUHCRHLIFEKTGAAIT--UQCT-ENLPSLCSUQGLRAE--ENVI
L.africana            291 LP---KDSELAPSSUCCHCRHLIFEKTGPAIT--UQCR-ENLPSLCSUQGLLAE--ENVI
E.telfairi            228 LS---RGSELGSRSUCUHCRHLIFEKAGSAIT--UQCE-EHLPTLCSUQGLWAE--ENVI
M.lucifugus           142 LP---RESESAASSUCUHCRHLVFEHTGSAIT--URCR-EALPSLCSULGLWAE--ENVI
C.familiaris          301 LP---RDSGLAPSSUCUHURHLIFEKTGSAIT--UQCK-ETLPSLCSUQGLWAE--ENVI
S.araneus             240 LP---KDSELAPSSUCUHCRHLVFEKTGSAIT--UQCN-ENLPSLCSUQGLWAE--ENVI
S.lateralis           268 LF---KDPQAASSSCCCHCRHLIFEKRESAIT--UQCA-ENLPSLCSUQGLRAE--ENVI
S.tridecemlineatus    249 LF---KDPQAASSSCCCHCRHLIFEKRESAIT--UQCA-ENLPSLCSUQGLRAE--ENVI
O.cuniculus           291 ML---KKSELAPSSCCCHCRHLIFENTGSAVT--URCR-ENLPSLCSUQGLLAE--ENVI
O.aries               274 FP---KDSESALSSCCCHCRHLVFEKTGSAIT--UQCT-ENLPSLCSUQGLLAE--ENVI
C.hircus                  ------------------------------------------------------------
B.taurus              294 FP---KYSESALSSCCCHCRHLVFEKTGSAIT--UQCT-EKLPSLCSUQGLLAE--ENVI
S.scrofa              298 FP---KNSESALRSUCUHCRHLIFEKTESAVT--UQCG-ENLPSLCSUQGLLAE--ENVI
R.norvegicus          294 LS---EESGAATSSCCCHCRHLIFEKSGSAIT--UQCA-ENLPSLCSUQGLFAE--EKVI
M.musculus            289 LS---KESEAAPSSCCCHCRHLIFEKSGSAIA--UQCA-ENLPSLCSUQGLFAE--EKVT
C.porcellus           225 PP---KNSHSAPSSCCCHCRHLVFEQPGSSVT--CQCA-ENLPSLCSCEGLFGE--EKVI
M.domestica           306 LS---QSSGSAPRSUCUHCRHLIFERLGNAVT--UHCKGTTLPSSCSUQGQRAE--ENIT
O.anatinus            297 WH---KRSGPAPSSUCUHCRHLLFGSKATATAL-UQCR-DALPALCSUQGRQSG--EDVI
G.gallus              296 WQ---TASDSTTSSUCCHCRHLLFEELGNSIT--UQCR-GALPNSCRUHGQLLA--EDIT
L.erinacea            204 --------GHGHES----------------------------P-----------------
S.acanthias               ------------------------------------------------------------
B.floridae            181 ----------------------PVPQETTPAPVITTAAPPTDTPVCAHPPSWQLDGVDHL
O.porcinus            178 -----------------------------------ATEAQRSTETSSANGTAAAPQVREG
B.microplus           175 RNPRNTDNPARRNSLGWRILHMFLGGEQDDESHECNERKHSQMRVC-CHPN--SHTES--
C.gigas               185 LQ---RQAVSSLSSIIRRRHVFNHFGTSLVSTNNVPSRNDRPSDU-------------PN
A.irradians           235 ER---NTPQSRHRMIHRLURHMRRRGFLGGSTHSUVCP-RNFRDVCSCTRYRQSRK---K
```

```
D.rerio              334 ETUQURPAPPAEU---------------ELSQPTUVUPAGDATUGURKK-----
C.carpio             244 ETUQURPAPPAEU---------------ELSQKTUAUPAGDASUVUKEK-----
I.punctatus          346 ETUQURPAHLGDU---------------QPAQPIUAUPAGISQUEUQVI-----
T.nigroviridis       348 ETUQURS-PLAVU--------------QQPQPAPUAUPQGAN-UGUEQV-----
T.rubripes           355 ETUQURS-PLAVU--------------QQPQPAQUAUPQGVN-UGUEQV-----
G.aculeatus          354 ETUQCRS-PPAAU--------------QQPEPAPUAUAAGVS-UGUEQL-----
S.salar              356 ETUQURS-PPADU--------------QQPPPVMUAUPLVES-UGUGQL-----
O.mykiss             246 ETUQURS-SLADU--------------QQPQPVMUAUPLGVESUGUGLL-----
Haplochromis sp.         ------------------------------------------------------
O.latipes            358 ESUQURS-PPAAU--------------QQPQPAQUAUAPGAASUAUEQL-----
F.heteroclitus       299 ETUQURM-PPAAU--------------QQPQPAQUAUPPGVGSUAUEQL-----
X.tropicalis_Cys     224 DHSQ-HHPINSHKRQENQNNHPRNLIKNGKQN----------------------
X.tropicalis         350 ESUQURL-SAAAUHSESTG--LPETKLESEPNAPUAUPQEAENUQUKELURFLM
X.laevis             345 ESUQURL-SAAAUHSHSTG--LPELDTESETNAPUAUPQEAENUQUKELURFLM
A.mexicanum          315 ESUQURV-SPAAUPSLAVDQLHEQLPSESDPNVAUQUPKATGTUQUQVLTN---
H.sapiens            341 ESCQURL-PPAAU-------QISQQLIPTEASASURUKNQAKKUEUPSN-----
P.troglodytes        341 ESCQURL-PPAAU-------QISQQLIPTEASTSUCUKNQAKKUEUPSN-----
P.pygmaeus           341 ESCQURL-PPPAU-------QISQQLIPTEASTSURUKNQAKKUEUPSN-----
M.mulatta            342 ESCQURL-PPAAU-------QISQQLVPTEVSTNURUKSKAKKUEUSSN-----
M.fascicularis       341 ESCQURL-PPAAU-------QISQQLVPTEVSTNURUKSKAKKUEUSSN-----
E.europaeus          203 ESUQURL-PPAAU------Q-SRQQLSPTTASATUSUNDKAGMUEUPSH-----
T.belangeri          166 ESCQURL-PPAAU------QT-SQQLSPTEVSTNUKUNKKAEEUKULSN-----
L.africana           343 ESCQURL-PPAAU-------QASQQLKPTGASTNUSUKNKAEMUKUPSN-----
E.telfairi           280 ESUQURL-PLPAU------QRS-QQFQPTEASTSUSUKNTAEKUKUPSN-----
M.lucifugus          194 ESUQURL-PPAAU--------QAGQLEPTEASTTUSUKTEAEMUQUPSN-----
C.familiaris         353 ESUQURW-PPAAU-------QASQQLRPTEASTNUSUKYKTKMUKULTY-----
S.araneus            292 ESUQURL-PPAAU-------QSGQQPKPTVDNTKUSUKNQAEMUKUPAN-----
S.lateralis          320 ESCQURM-PPAAU-------QRSQQLDPTEVGTNUSUKNKAKKUKUHSN-----
S.tridecemlineatus   301 ESCQURM-PPAAU-------QRSQQLDPTEVGTSUSUKNKAKKUKUHSN-----
O.cuniculus          343 ESCQURL-PPAAU-------QTSQQLKPTEASTNUSUNNLAKKUKUPSN-----
O.aries              326 ESUQURL-PPAAU------QAAGQQLNPTEASTKUSUKNKAKMUKUPSN-----
C.hircus                 ------------------------------------------------------
B.taurus             346 ESUQURL-PPAAU------QAAGQQLNPTEASTKUSUKNKAKMUKUPSN-----
S.scrofa             350 ESUQURL-PPAAU------QAS-QQLNPAEASTKUSUKNKAGRUKUPSN-----
R.norvegicus         346 ESCQCRS-PPAAU--------HSQHVSPTEASPNUSUNNKTKKUKUNLN-----
M.musculus           341 ESCQCRS-PPAAU--------QNQPMNPMEANPNUSUDNQTRKUKUHSN-----
C.porcellus          277 ESCQCRL-PLAPU-------QVSPQQKPTETNPNUSUKNMAQKUKUPSN-----
M.domestica          359 ESUQURS-PPAAU-------HPGQQLEPTETRNAUKUKNNAGRUKUSTN-----
O.anatinus           350 ESUQURS-PLPAU----PPAAQLPSPSPTDPNAAUKUENTAGMUKUPTR-----
G.gallus             348 ESUQURL-LTAAU--------ESAAGGGSETSDTUQUQERAGNUAUKTN-----
L.erinacea               ------------------------------------------------------
S.acanthias              ------------------------------------------------------
B.floridae           219 EKSQGQ------------------------------------------------
O.porcinus           203 ESPEPRHNGV--------------------------------------------
B.microplus          215 PYCR----QAPSC------------EELGAACGSU-UQ----------------
C.gigas              230 SRURRQLSNIVGTQKSVPS-----------------------------------
A.irradians          288 RFCACSEI----------------------------------------------
```

Figure S2. Multiple sequence alignment of Selenoprotein P sequences. Cysteine residues are highlighted in blue, and selenocysteine residues in red. Sequences with the following accession numbers were used generate the alignment:
H. sapiens (NP_001087195.1), *P. pygmaeus* (CAH91791.1), *D. rerio* (AAG53688.1), *B. taurus* (BAA84781.1), *R. norvegicus* (NP_062065.2), *M. musculus* (NP_033181.3), *C. familiaris* (XM_862927.1), *G. gallus* (NP_001026780.2), *M. domestica* (AAFR03031319.1), *P. troglodytes* (XM_530812.2), *S. scrofa* (AJ945481.1, DB795066.1), *M. mulatta* (XM_001087397.1), *M. fascicularis* (AB169844.1), *O. aries* (CO202513.1, CN823413.1), *O. cuniculus* (DN889827.1, EC620121.1), *C. hircus* (AAF67201.1), *O. anatinus* (XR_036552.1, XM_001519849.1), *X. tropicalis* (BC084988.1), *X. tropicalis* Cys homolog

(NM_001006907.1), *X. laevis* (BC128687.1), *S. lateralis* (CO739434.1), *S. tridecemlineatus* (ES429752.1, ES430681.1), *T. nigroviridis* (CR655078.2), *G. aculeatus* (BT027180.1), *S. salar* (DW536174.1, CA045197.1), *B. microplus* (CV452517.1), *A. mexicanum* (CN034653.1, CO790560.1), *O. latipes* (BJ910837.1, BJ734131.1), *O. mykiss* (CX255200.1, CA375171.1), *C. carpio* (CA966337.2, CF662894.2), *T. belangeri* (AAPY01533128.1, AAPY01533129.1), *E. europaeus* (AANN01197889.1), *M. lucifugus* (AAPE01210967.1, AAPE01518317.1), *E. telfairi* (AAIY01264272.1, AAIY01707893.1), *L. africana* (AAGU01358562.1, AAGU01358563.1), *C. porcellus* (AAKN01329275.1, AAKN01257578.1, AAKN01257577.1), *S. araneus* (AALT01420282.1, AALT01420283.1), *F. heteroclitus* (CN981462.1, CN980688.1), *L. erinacea* (CO051159.1), *O. porcinus porcinus* (CB722105.1), *T. rubripes* (CAAB01001108.1), *I. punctatus* (EE993430.2, CK420078.1), *A. irradians* (CK484474.1), *C. gigas* (AJ565579.1), *B. floridae* (BI386612.1).

Figure S3

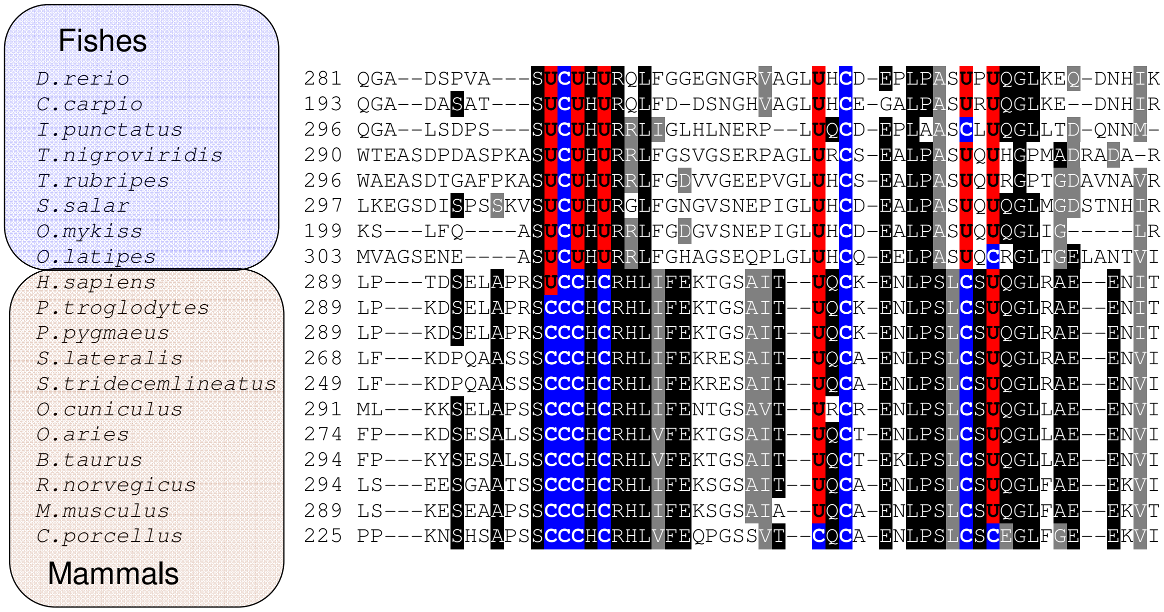

Figure S3. Partial alignment of fish and mammalian SelP sequences. Sec residues are highlighted in red and Cys residues in blue. Loss of Sec is shown by a box.

Figure S4

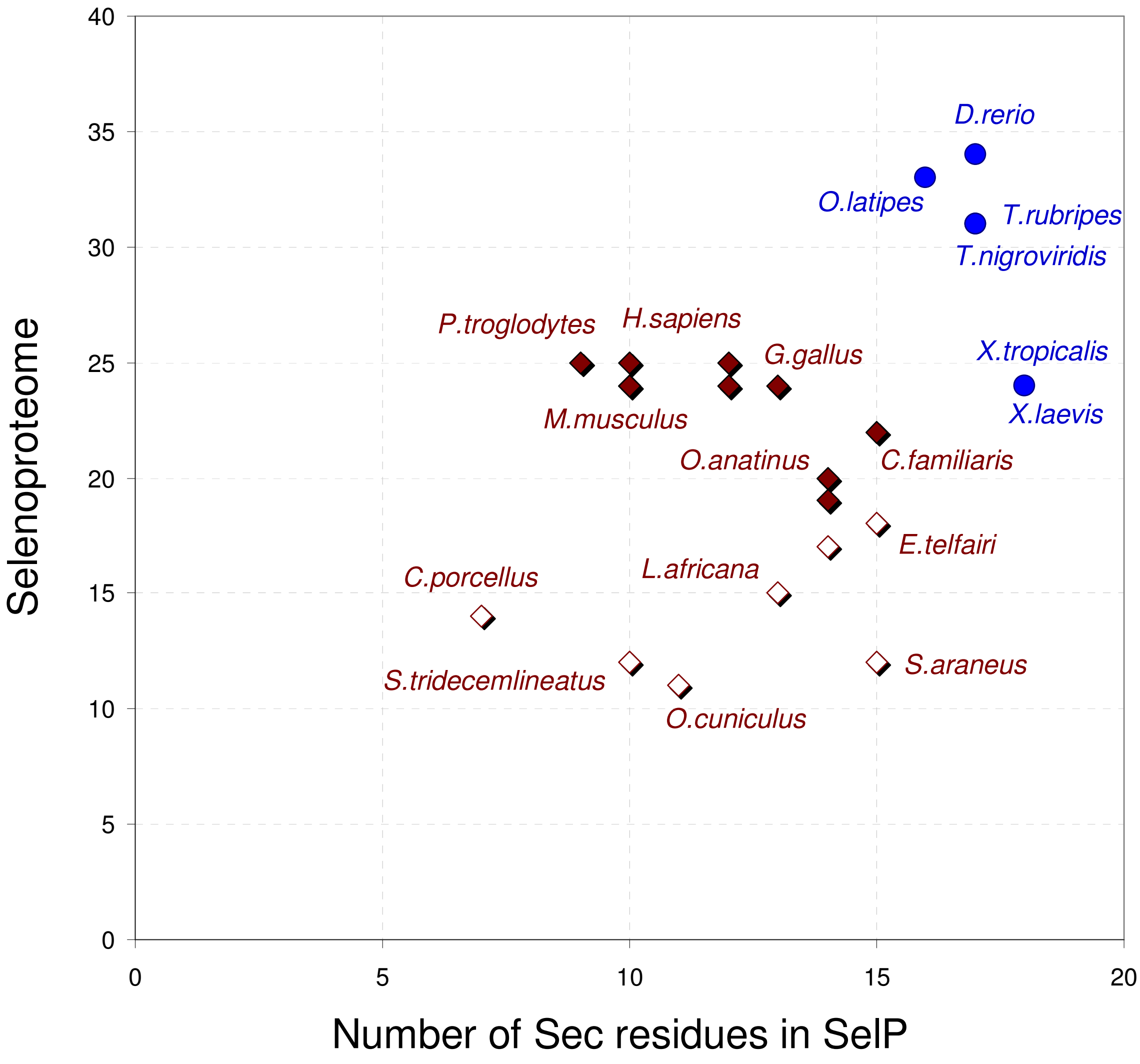

Figure S4. A plot of selenoproteome size versus Sec content of SelPs. A 2D-representation of the relationship between the number of Sec residues in SelPs and selenoproteome size in vertebrates is shown. The data for aquatic vertebrates (including fish and amphibians) are shown in blue, and terrestrial (mammals and birds) in brown. Open squires and circles correspond to genomes with 1.7x-2.3x coverage, and filled to genomes with 5x-9x coverage.

Figure S5

A. Selenocysteine loss in primates

H. sapiens	289	LPTDSELAPRSUCCHCRHLIFE	310
M. mulatta	289	LPTDSELAPRSUCCHCRHLIFE	310
P. troglodytes	289	LPTDSELAPRSCCCHCRHLIFE	310
P. pygmaeus	290	LPTDSELAPRSCCCHCRHLIFE	311

B. Selenocysteine loss in fish

T. rubripes	334	EALPASUQURGPTGDAVNAVR	354
S. salar	335	EALPASUQUQGLMGDSTNHIR	356
O. mykiss	230	EALPASUQUQGLIG-----LR	245
O. latipes	337	EELPASUQCRGLTGELANTVI	357

C. Selenocysteine switch in *Xenopus*

X. tropicalis	325	ENEVAUHUQEALPSSCSUQELL	346
X. laevis	318	QNEVAURCQEALPASUQUQELL	339

Figure S5. Recent Sec/Cys conversions in closely related species. A. Sec loss in primates. Partial amino acid sequence alignment is shown, with Sec residues highlighted in red and Cys residues in blue. Loss of Sec is indicated by a box. **B. Selenocysteine loss in fish.** Partial amino acid sequence alignment is shown. Sec and Cys residues are highlighted in red and blue, respectively. Box indicates the loss of Sec. **C. Sec switch in *Xenopus*.** Partial amino acid sequence alignment is shown, with Sec highlighted in red and Cys in blue. The number of Sec and Cys residues is the same in SelPs from both *Xenopus* species, but the locations of the two indicated Sec residues are different, as shown by boxes.

Figure S6

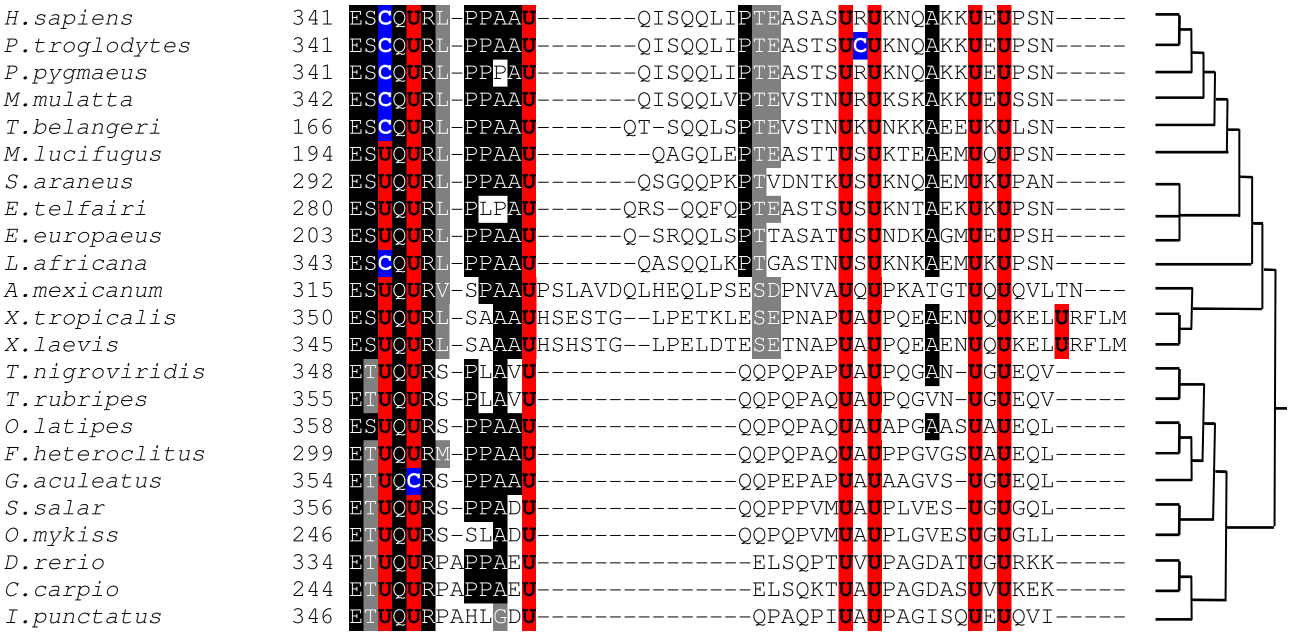

Figure S6. Evolution of new Sec residues by C-terminal extension. *Xenopus* SelP is extended at the C-terminus as shown here by partial alignment of C-terminal sequences of SelPs from indicated organisms. Sec residues are highlighted in red, and Cys residues in blue. The new Sec in *Xenopus* SelP corresponds to stop codons in most other vertebrate SelPs.

Figure S7

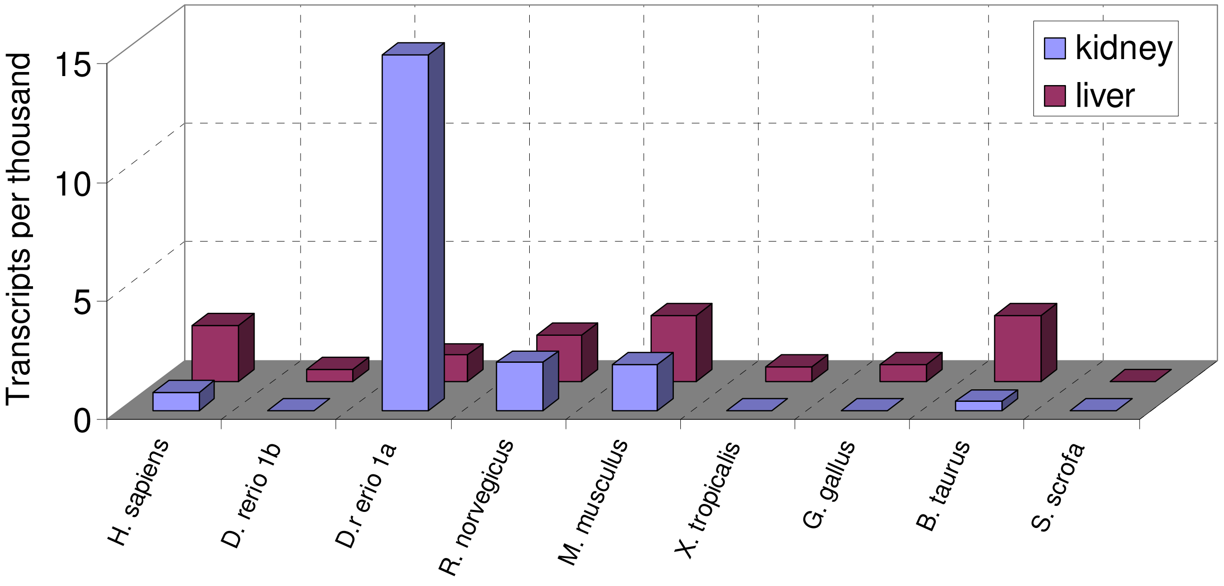

Figure S7. *In silico* expression profile of SelP based on abundance of SelP ESTs. Expression levels in kidney and liver for SelPs from different organisms are shown in transcripts per million. UniGene's EST ProfileViewer was used in this analysis.

Figure S8

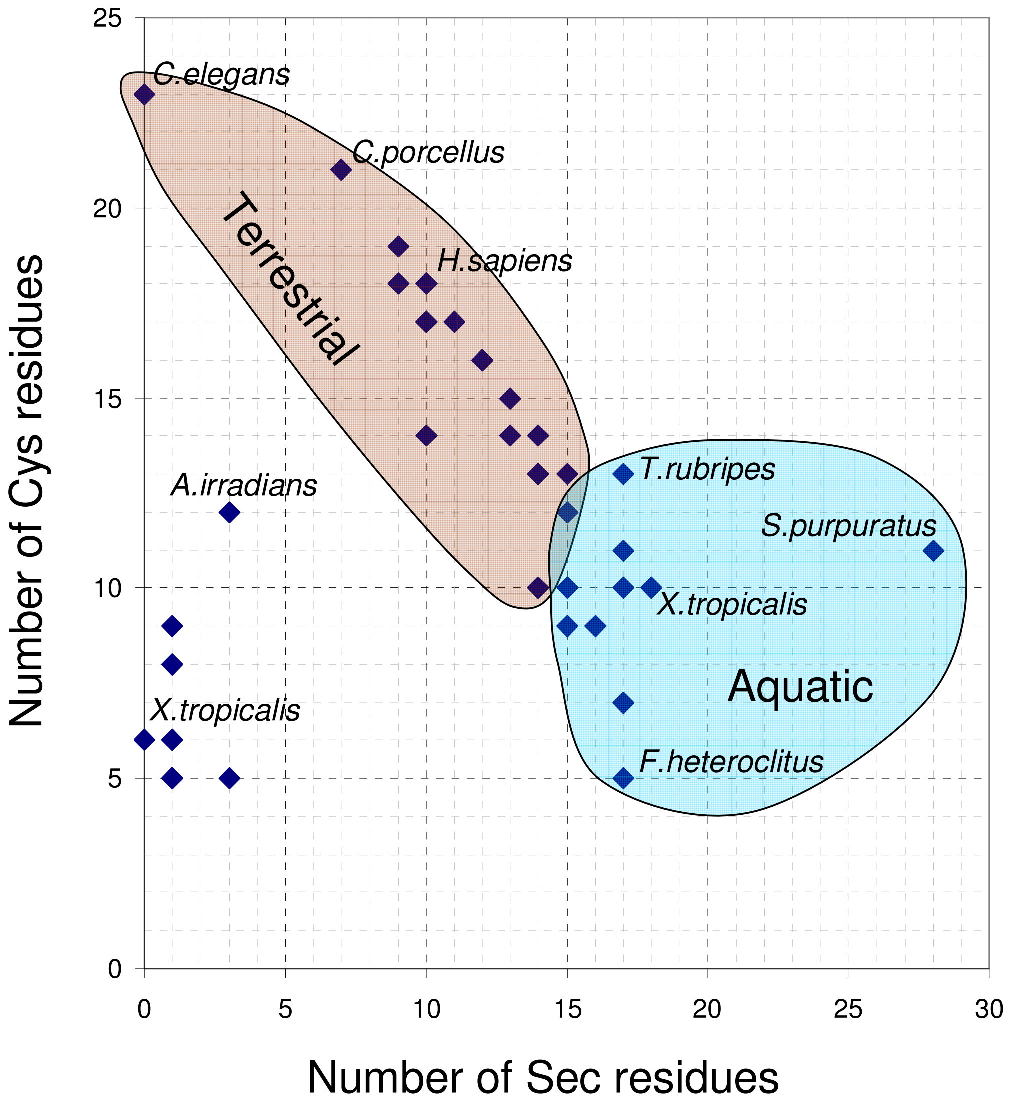

Figure S8. Correlation between Cys and Sec content of SelPs. A 2D-representation of Cys and Sec distribution in SelPs from different organisms is shown. Star symbols indicate that sequences used in analysis are incomplete, and the real number of Sec or Cys residues most likely is higher. Blue area highlights aquatic organisms and brown terrestrial SelP sequences containing a C-terminal Se transport domain. The data points outside of these areas show single-Sec SelPbs that lack the C-terminal Se transport domain.
